# The Parkinson’s Disease-Associated Protein DJ-1 Protects *Dictyostelium* Cells from AMPK-Dependent Outcomes of Oxidative Stress

**DOI:** 10.3390/cells10081874

**Published:** 2021-07-23

**Authors:** Suwei Chen, Sarah J. Annesley, Rasha A. F. Jasim, Paul R. Fisher

**Affiliations:** 1 Discipline of Microbiology, Department of Physiology Anatomy and Microbiology, School of Life Sciences, La Trobe University, Bundoora, VIC 3086, Australia; chensuwei@aku.edu.cn (S.C.); S.Annesley@latrobe.edu.au (S.J.A.); sawa_mm2003@yahoo.com (R.A.F.J.); 2 School of Modern Agriculture and Biotechnology, Ankang University, Ankang 725000, China; 3 Department of Laboratory and Clinical Sciences, College of Pharmacy, University of Babylon, Hillah 51002, Iraq

**Keywords:** Parkinson’s disease, DJ-1, PARK7, AMPK, ROS, oxidative stress, *Dictyostelium*

## Abstract

Mitochondrial dysfunction has been implicated in the pathology of Parkinson’s disease (PD). In *Dictyostelium discoideum*, strains with mitochondrial dysfunction present consistent, AMPK-dependent phenotypes. This provides an opportunity to investigate if the loss of function of specific PD-associated genes produces cellular pathology by causing mitochondrial dysfunction with AMPK-mediated consequences. DJ-1 is a PD-associated, cytosolic protein with a conserved oxidizable cysteine residue that is important for the protein’s ability to protect cells from the pathological consequences of oxidative stress. *Dictyostelium* DJ-1 (encoded by the gene *deeJ*) is located in the cytosol from where it indirectly inhibits mitochondrial respiration and also exerts a positive, nonmitochondrial role in endocytosis (particularly phagocytosis). Its loss in unstressed cells impairs endocytosis and causes correspondingly slower growth, while also stimulating mitochondrial respiration. We report here that oxidative stress in *Dictyostelium* cells inhibits mitochondrial respiration and impairs phagocytosis in an AMPK-dependent manner. This adds to the separate impairment of phagocytosis caused by DJ-1 knockdown. Oxidative stress also combines with DJ-1 loss in an AMPK-dependent manner to impair or exacerbate defects in phototaxis, morphogenesis and growth. It thereby phenocopies mitochondrial dysfunction. These results support a model in which the oxidized but not the reduced form of DJ-1 inhibits AMPK in the cytosol, thereby protecting cells from the adverse consequences of oxidative stress, mitochondrial dysfunction and the resulting AMPK hyperactivity.

## 1. Introduction

Autosomal, recessive point mutations and deletions in the human DJ-1 gene (PARK7) cause a form of familial Parkinson’s disease (PD) accounting for 1–2% of early onset cases [1,2,3]. Various functions of DJ-1 have been proposed, including roles as a redox-sensitive chaperone [4,5], a transcriptional regulator [6,7] and an active protease [8,9] contributing to protein homeostasis within the cells. Another function that has been proposed for DJ-1 is as an antioxidant to protect the cells under oxidative stress [10,11,12,13]. In addition, Blackinton et al. [14] reported that DJ-1 has a mitochondria-stabilizing role, while Larsen et al. [15] showed that DJ-1 knockdown impaired astrocyte mitochondrial motility and fusion and reduced the mitochondrial membrane potential. DJ-1 also interacts with other PD-linked genes, including *PINK1* and *SNCA* (encoding α-synuclein), which have been reported to affect mitochondrial function [16,17]. These findings suggest that DJ-1 may function as a protector or positive regulator of mitochondrial function, a role it could exert either indirectly or directly in the mitochondria.

Whether or not DJ-1 localizes to the mitochondria has been a controversial issue. The DJ-1 protein sequence does not include a recognizable targeting signal directing it to either the mitochondria or the nucleus [18]. Bonifati et al. [2] transfected Cos and PC12 cells and found that wild type DJ-1 localized to the cytoplasm and nucleus. According to Zhang et al. [19], however, DJ-1 localizes to the mitochondrial matrix and intermembrane space in mouse brain tissues and human neuroblastoma cells. Kojima et al. [20] found that certain DJ-1 mutant forms localized to the mitochondria, whereas the wild type and other mutant forms did not. This question is made more complicated by the studies suggesting that oxidative stress causes wild type DJ-1 to relocalize from its usual cytosolic location to the mitochondria [10]. Yet, according to Björkblom et al. [21], the wild-type DJ-1 translocates from the cytosol to the nucleus after oxidative stress-induced monomerization, whereas after the monomerization of wild type/E163K heterodimers, it was the mutant E163K monomer that relocated to the mitochondria. Thus, the functional interactions between DJ-1, the mitochondria and oxidative stress and their roles in PD cytopathology remain poorly understood.

We recently showed that DJ-1 is located in the cytosol of the social amoeba *Dictyostelium discoideum*, regardless of whether or not the cells are exposed to oxidative stress [18]. *D. discoideum* is one of 10 valuable non-mammalian models for biomedical research according to the US National Institutes of Health and its genome includes a single gene, deeJ, encoding a homologue of human DJ-1 [22,23,24,25,26]. Its unique life cycle, reproducible genotype–phenotype correlations, genetic tractability and the simplicity of gene manipulation reduce the complexity of studying disease-associated signaling pathways in higher animals or in humans. The *Dictyostelium* mitochondrial disease model is well established, with genetically created mitochondrial dysfunction producing consistent, phenotypic outcomes [24] and the mitochondrial respiratory function being readily measured [27]. In unstressed cells, *Dictyostelium* DJ-1 plays a positive nonmitochondrial role in phagocytosis and its loss does not phenocopy mitochondrial dysfunction [18]. Thus, DJ-1 knockdown impairs the phagocytic uptake and growth on bacteria, has only slight effects on macropinocytosis and growth in liquid medium and no effect on phototaxis, but causes thickened stalks in the fruiting bodies. Furthermore, a reduced expression of DJ-1 causes an elevation rather than an impairment of mitochondrial respiration, thereby mimicking the mitochondrial hyperactivity recently observed in lymphoblasts from idiopathic PD patients [28] and fibroblasts from patients with Parkin mutations [29]. However, the functions of DJ-1 could be different under oxidative stress conditions, and it is this question that we addressed in the present study.

Similar to its human counterpart [10], *Dictyostelium* DJ-1 contains a conserved cysteine residue that, in the human protein, is oxidized under oxidative stress conditions. Although this does not cause DJ-1 to translocate to the mitochondria [18], it could nonetheless alter the protein’s activity so that, in combination with oxidative stress, its loss could impairs mitochondrial respiration. Here, we report that this in fact is not the case. Instead, oxidative stress and DJ-1 loss exert opposite, independent (additive) effects on mitochondrial function, whereby oxidative stress inhibits and DJ-1 loss enhances oxidative phosphorylation rates.

Previous reports that DJ-1 protects the mitochondria under oxidative stress did not measure mitochondrial respiratory function directly, but instead measured the downstream cytopathological consequences of mitochondrial dysfunction. Most of the phenotypic outcomes displayed by mitochondrially diseased *Dictyostelium* strains are mediated by chronic hyperactivity of the energy-sensing protein kinase AMPK [24,30]. AMPK is activated by a variety of cellular stresses, including ATP depletion (elevated AMP/ADP to ATP ratios) and oxidative stress. It homeostatically inhibits diverse energy-consuming pathways, including growth, while activating the energy-producing catabolism and mitochondrial biogenesis. Here, we show that the combination of oxidative stress and DJ-1 loss causes more severe phenotypic outcomes than either alone and that the additional effects of oxidative stress in DJ-1 knockdown cells are AMPK-dependent. Together, our results support a model whereby DJ-1 not only plays a primary, positive role in specific endocytic pathways and inhibits mitochondrial respiration [18] but, under oxidative stress conditions, also inhibits AMPK, thereby protecting the cells from AMPK-mediated cytopathological consequences.

## 2. Materials and Methods

### 2.1. Plasmid Constructs

The antisense constructs contained gene fragments cloned in the antisense orientation with respect to the actin 6 promoter in the vector pDNeo2 [31]. The DJ-1 antisense construct pPROF688 contains nucleotides 75–479 of the DJ-1 gene [18]. The AMPK antisense construct pPROF362 contains nucleotides 364–1536 of the AMPK α subunit gene [30]. The DJ-1 overexpression construct pPROF690 contains the full length DJ-1 gene expressed under the control of the actin 15 promoter in the vector pPROF267 [18].

### 2.2. D. discoideum Strains and Culture Conditions

The culture conditions of *D. discoideum* cells during growth on solid and liquid mediums were as described previously [18]. *D. discoideum* is well known as having a high level of resistance to reactive oxygen species such as H_2_O_2_, presumably because its natural environment in soil and its phagocytic lifestyle often expose it to high levels of these and other toxic agents. Others have found that concentrations of cumene hydroperoxide or H_2_O_2_ in a low millimolar or high micromolar range are required to kill wild type *Dictyostelium* cells [32,33]. Thus, exposure of growth phase cells to 500 µM for 60 min killed only 5% of the cells, while exposure to 5 mM H_2_O_2_ for the same time period killed 95% of the cells [32]. Similarly, a 24-h exposure to 500 µM killed only 33% of growth phase cells [33]. Although the mechanisms of this resistance are not fully understood, they do involve expression of endogenous antioxidant enzymes including catalase; therefore, *catA* mutants lacking the growth phase catalase are two orders of magnitude more sensitive than wild type cells to H_2_O_2_ [32].

In this work, all experiments were conducted with *D. discoideum* wild type parental strain AX2 and transformants derived from it [34]. The DJ-1 antisense and overexpression transformants were described previously [18]. Strains HPF1164–HPF1179 carried multiple copies of the DJ-1 antisense inhibition construct pPROF688; strains HPF1206–HPF1219 carried multiple copies of the DJ-1 overexpression construct pPROF690. The DJ-1/AMPK double antisense transformants were isolated in this work—strains HPF1180–HPF1190 carried multiple copies of both the DJ-1 antisense construct pPROF688 and the AMPK antisense construct pPROF362.

### 2.3. Molecular Manipulation of DNA and RNA

#### 2.3.1. Quantitative PCR (qPCR)

The construct copy numbers in *D. discoideum* transformants was quantitated by quantitative PCR by using iQ SYBR Green Supermix as instructed by the manufacturer (Bio-Rad, Hercules, CA, USA) and described previously [18].

#### 2.3.2. One-Time Reverse Transcriptase-PCR (RT-PCR)

The RNA was quantitated using an iScript^TM^ One-Step RT-PCR Kit (Biorad) described previously [18].

### 2.4. Cotransformation of Dictyostelium

The cotransformation of *Dictyostelium* was conducted as described previously for the transformation of *Dictyostelium*, using the Ca(PO_4_)_2_/DNA coprecipitation method developed by Nellen et al. [35], except that a mixture of 2 antisense inhibition constructs (in this case pPROF688 for DJ-1 antisense inhibition and pPROF362 for AMPK antisense inhibition) was used in the transformation. The cotransformants were isolated by selection and growth on *Micrococcus luteus* lawns on nutrient agar (Standard Medium, SM) supplemented with 20 μg mL^−1^ G418, as described by Wilczynska and Fisher [36].

### 2.5. Phenotypic Characterization

#### 2.5.1. Seahorse Respirometry

The method was described in detail by Lay et al. [27] and is illustrated in Supplementary Figure S1. The exponentially growing *Dictyostelium* amoebae in HL-5 medium with or without 450 µM H_2_O_2_ were harvested, washed and resuspended in SIH assay medium (Formedium, Hunstanton, Norfolk, United Kingdom) supplemented with 20 mM sodium pyruvate and 5 mM sodium malate (pH 7.4). For each strain to be tested, 1 × 10^5^ cells were inoculated into each of 8 Matrigel-coated wells in a 24-well assay plate for the Seahorse XFe24 Flux Analyzer and allowed to attach. After the calibration and equilibration steps, measurements throughout the assay were conducted using cycles of 3 min mixing, 2 min wait and 3 min measurement time. The basal O_2_ Consumption Rate (OCR) was measured for 3 measurement cycles and this was followed by OCR measurements after sequential injections of 10 µM N,N′-dicyclohexylcarbodiimide (DCCD, ATP synthase inhibitor, Sigma-Aldrich (Merck Pty. Ltd., Bayswater, Melbourne, Australia); 6 measurement cycles), 10 µM carbonyl cyanide 3-chlorophenol hydrazone (CCCP, protonophore, Sigma-Aldrich; 3 measurement cycles), 20 µM rotenone (Complex I inhibitor, Sigma-Aldrich; 3 measurement cycles) and either 10 µM antimycin A (Complex III inhibitor, Sigma-Aldrich; 4 wells, 3 measurement cycles) or 1.5 mM benzohydroxamic acid (Alternative Oxidase/AOX inhibitor, BHAM, Sigma-Aldrich; 4 wells, 3 measurement cycles). The parental AX2 strain was included in every experiment in 4 wells (2 for each of the final antimycin A and BHAM injections).

#### 2.5.2. Phototaxis

A 1 cm^2^ area in the center of each water agar plate was inoculated with 2.5 × 10^6^ amoebae and the phototaxis assay was performed as described previously using water agar with or without 900 µM H_2_O_2_ [18,36,37]. Trails were digitized using a Summagraphics 120 digitizing tablet hosted on a Linux computer and replotted from a single starting point as previously described [37].

#### 2.5.3. Growth in Axenic Liquid Medium

Growth in HL-5 medium was assayed as previously described [18,30]. Amoebae from an exponentially growing culture in HL-5 medium were inoculated into 50 mL of HL-5 with or without 450 µM H_2_O_2_. The concentration of H_2_O_2_ selected was determined separately to be sufficient to reduce the growth rate of AX2 by 50% (IC50). Cell counts were determined twice daily for 5 days using a hemocytometer, and lag phase and generation times were determined using custom software in the R statistical and graphics package (version 4.1.0). The generation time was calculated from the slope of the line in log-linear regression of the cell counts against the time during the exponential phase of growth. The lag phase was determined as the time at which the linear portion of the log-linear growth curve intercepted the starting cell count.

#### 2.5.4. Morphogenesis

Fruiting body morphology on phosphate buffered agar (KK2) plates. Amoebae were inoculated onto 1 cm^2^ areas on KK2 agar plates at a density of 5 × 10^6^ cells/cm^2^. For exposure to oxidative stress, the agar was supplemented with 900 µM H_2_O_2_. Plates were incubated at 21 °C for a week and the fruiting body morphology was observed and photographed as previously described [18,30,38].

Fruiting body morphology on SM plates. Amoebae from the edge of colonies growing on SM agar plates on *Enterobacter aerogenes* lawns were streak-diluted onto fresh SM plates that had been supplemented with 900 μM H_2_O_2_ and covered with a thick, fully pregrown lawn of *E. aerogenes*. These plates were incubated at 21 °C until fruiting bodies were visible. For oxidative stress, the SM agar was supplemented with 900 µM H_2_O_2_. The fruiting body morphology was observed and photographed as previously described [18,30,38].

#### 2.5.5. Phagocytosis and Pinocytosis

*D. discoideum* strains were grown in HL-5 media containing 450 μM H_2_O_2_ to a density of 1–2 × 10^6^. Phagocytosis was measured as the rate of uptake of DS-Red-expressing fluorescent *E. coli* B cells and pinocytosis as the rate of uptake of FITC-dextran-containing liquid medium. Phagocytosis and pinocytosis experiments were carried out as described previously [18,30,39] with the exception that media, solutions and buffers used for imposition of an oxidative stress contained 450 μM H_2_O_2_.

### 2.6. Statistical Analysis

Statistical analysis was conducted by standard methods using WinSTAT addon software (version 2012.1.0.96, R. Fitch Software) for Microsoft Excel, including multiple regression analysis with dummy variables, *t*-tests of the significance of regression coefficients and F tests of the significance of an overall regression.

## 3. Results

### 3.1. Oxidative Stress Impairs Mitochondrial Respiration Independently of DJ-1

Canet-Aviles et al. [10] showed that in human cells oxidatively stressed by exposure to the oxidant paraquat, C106 in DJ-1 was oxidized to cysteine sulfinic (SO3) acid. DJ-1 has been shown to protect cells from the cytopathological consequences of oxidative stress in a manner that depends upon its oxidation at C106 [10,11,12,13], a conserved cysteine residue whose counterpart in *Dictyostelium* is C117 [18]. The preliminary Western blotting results using a commercial antibody directed against this sulphonylated cysteine in the human protein suggested that similar to its human counterpart, *Dictyostelium* DJ-1 is also oxidized by H_2_O_2_ at this residue (Supplementary Figure S2). H_2_O_2_ is a strong oxidant and commonly used to create oxidative stress in mammalian cells [40,41].

**Figure 1:**
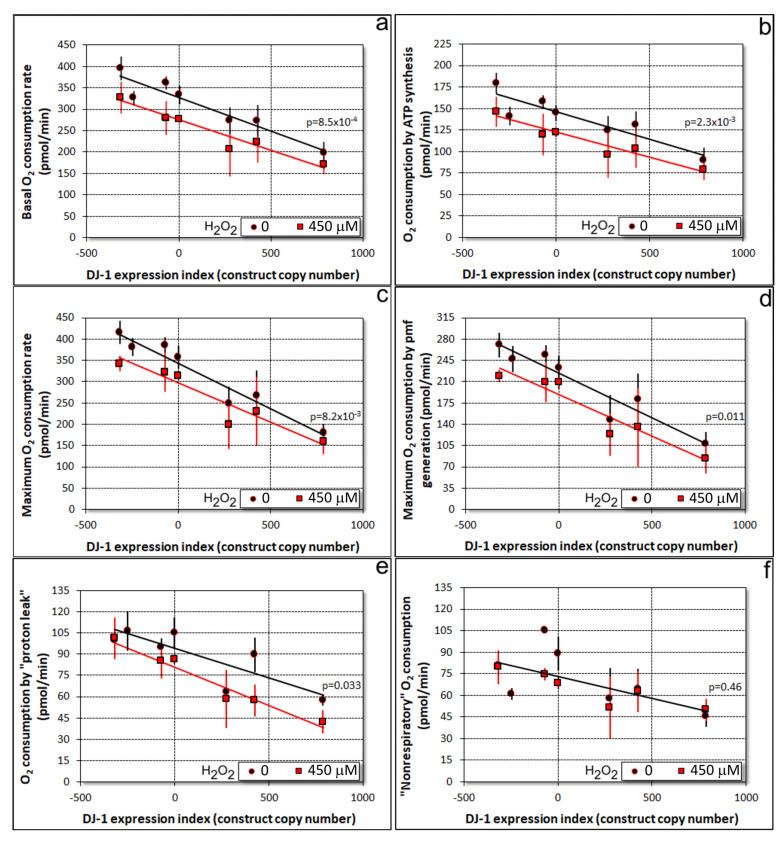
Mitochondrial respiratory activity is inhibited separately by oxidative stress and DJ-1. O_2_ consumption rates (OCR) in respiration were measured for 1 × 10^5^ cells per well in a Seahorse XFe^24^ Extracellular Flux Analyzer. An explanatory example trace is shown in Supplementary Figure S1. After measurement of basal respiration (**a**), the OCR was then measured after successive additions of ATP synthase inhibitor oligomycin (the resulting decrease representing OCR by ATP synthesis (**b**)), the proton ionophore CCCP (to uncouple respiration, allowing maximum O_2_ consumption (**c**)), the Complex I inhibitor rotenone and either the Complex III inhibitor antimycin A or the Alternative Oxidase inhibitor BHAM (the sum of the decreases caused by these latter 3 inhibitors representing the maximum pmf-generating OCR *ie.* OCR by proton pumping (**d**)). The “nonrespiratory” OCR (**f**) was calculated as the maximum OCR minus the pmf-generating OCR) and the OCR by the “proton leak” (**e**) was calculated as the difference between the OCR after oligomycin addition and the “nonrespiratory” OCR. Multiple regression analysis was conducted to assess the significance of effects of oxidative stress (exposure to 450 μM H_2_O_2_) and altered DJ-1 expression levels. The DJ-1 expression index for each individual transformant was determined using qPCR and expressed using the previously established convention of assigning negative copy numbers to the antisense inhibition (knockdown) and positive numbers to the overexpression constructs [18]. The constructs are inserted stably at one or a few random sites in the *Dictyostelium* genome where they are tandemly replicated during the integration process [42]. The insertion sites and copy numbers differ in each individual transformant. Each point represents a single, independent transformant strain assayed in 4 separate wells (technical replicates) per experiment and averaged over 3 independent experiments. All electron transport-driven components of respiration were inhibited to a proportionately similar extent, both by DJ-1 and by oxidative stress. The significance probabilities shown represent the probability that the intercepts of the regression lines are the same, with and without oxidative stress. They, therefore, represent the statistical significance of the inhibition of mitochondrial function by H_2_O_2_. Error bars are standard errors of the mean.

The protective role for DJ-1 under conditions of oxidative stress is commonly interpreted to imply protection against the impairment of mitochondrial respiratory function. If true, then the presence of more oxidizable DJ-1 would result in less inhibition of mitochondrial respiration by oxidative stress and vice versa. We were able to test this hypothesis using a Seahorse XFe24 Extracellular Flux Analyzer to assay the mitochondrial respiratory function in DJ-1 knockdown and overexpression strains, in the presence and absence of oxidative stress. The results (Figure 1) showed that growth-inhibiting concentrations of H_2_O_2_ inhibit mitochondrial respiration to an extent that is the same across a wide range of DJ-1 expression levels (red lines parallel to, but significantly below, the black lines in Figure 1a–e). Rather than protecting and enhancing mitochondrial respiration in oxidatively stressed cells, DJ-1 inhibits mitochondrial respiration in a gene expression-dependent manner exactly as in unstressed cells. The magnitude of the effect of exposure to 450 µM H_2_O_2_ was constant, regardless of the extent of DJ-1 antisense inhibition; therefore, the slope of the line relating the DJ-1 expression index to OCR was the same with or without H_2_O_2_ exposure. Thus, the effect of oxidative stress and DJ-1 expression were additive and independent, each being unaffected by the other.

Similar outcomes were found for basal respiration (Figure 1a) and both of its major electron transport-driven components, ATP synthesis (Figure 1b) and the so-called “proton leak” (Figure 1e). The latter represents the use of the proton-motive force (pmf) for purposes other than ATP synthesis, such as protein import, ion and metabolite exchanges across the mitochondrial membrane. In mitochondria uncoupled by treatment with the ionophore CCCP, oxidative stress and DJ-1 expression levels also combined in similar ways to affect the maximum uncoupled respiration rate (Figure 1c) and its pmf-generating components (Figure 1d), the combined activities of Complex I and II, coupled to Complexes III and IV.

The only exception was that oxidative stress did not significantly affect oxygen consumption by other cellular oxidases and oxygenases (referred to as “nonmitochondrial” or “nonrespiratory” O_2_ consumption) (Figure 1f). This component of oxygen consumption can be regarded as a broadly representative surrogate for the rates of nonmitochondrial cellular metabolism. Although inhibited by DJ-1 (similar to other components of respiration), it was unaffected by H_2_O_2_. Accordingly, this result indicates that the primary site of action of oxidative stress on metabolism in these cells is in the mitochondria.

### 3.2. Under Oxidative Stress, Knockdown of DJ-1 Produced AMPK-Mediated Phenotypic Defects in D. discoideum

The foregoing results suggest that loss-of-function, PD-causing mutations in DJ-1 would not exacerbate the inhibition of mitochondrial respiration by oxidative stress but, if anything, ameliorate it. Yet, it is well established that DJ-1 loss does exacerbate some of the downstream consequences of oxidative stress on the mitochondria, partly via interactions with two other PD-associated proteins, PINK1 and Parkin [43]. PINK1 is a partly mitochondrial protein kinase and Parkin a cytosolic ubiquitin ligase that translocate to and interact at the mitochondrial outer membrane under oxidative stress. This raises the question of whether in the *Dictyostelium* model, DJ-1 loss (knockdown) exacerbates or ameliorates the downstream phenotypes arising from the mitochondrial dysfunction caused by oxidative stress.

Mitochondrial dysfunction in *Dictyostelium* produces an array of well characterized, reproducible phenotypic outcomes, mostly mediated by the chronic hyperactivation of AMPK [24,30]. In unstressed *Dictyostelium* cells, the phenotypic consequences of DJ-1 loss (knockdown) are distinct from those caused by mitochondrial dysfunction and AMPK hyperactivity [18]. If DJ-1 loss exacerbates the effects of mitochondrial dysfunction under conditions of oxidative stress, these phenotypes should become apparent during exposure to H_2_O_2_. To investigate this, DJ-1 antisense-inhibited transformants were characterized phenotypically after and during exposure to H_2_O_2_.

**Figure 2:**
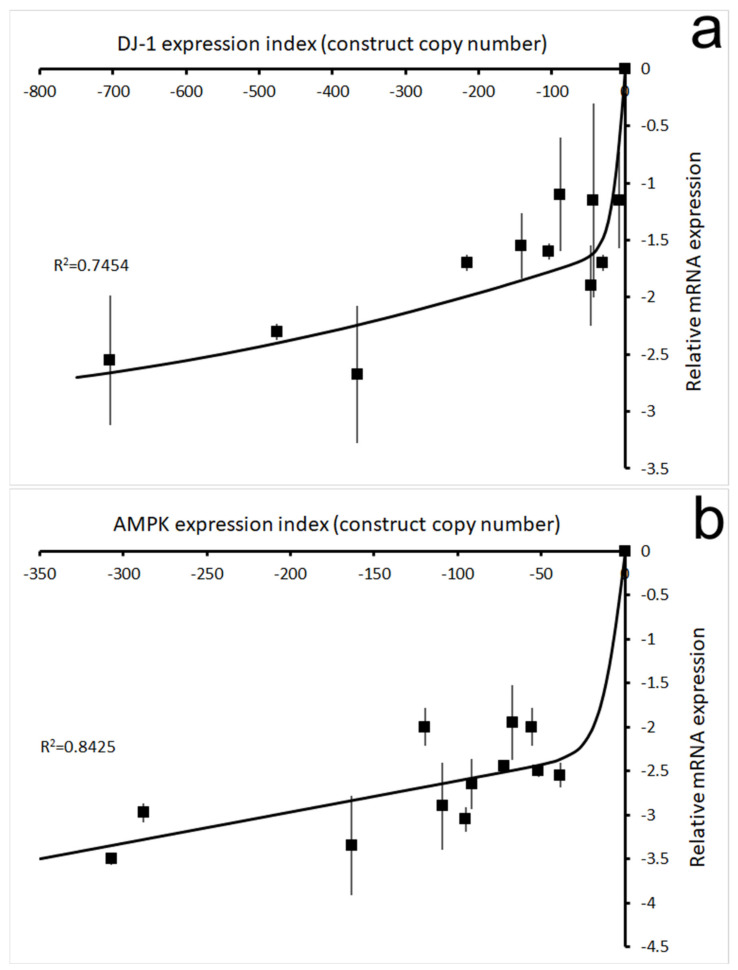
Correlation of construct copy numbers with mRNA expression levels in cotransformant strains (cell lines) containing *both* pPROF688 (DJ-1 antisense construct) and pPROF362 (AMPK antisense construct). The mRNA levels (Y axis) were measured in qRT-PCR cycle number differences from those for the filamin gene (the loading control reference gene) and then expressed as the difference from the level in the parental strain AX2 (which, therefore, has a relative expression level of 0). (**a**): The relationship between DJ-1 antisense inhibition construct (pPROF688) copy numbers and DJ-1 mRNA levels. The figure shows the coefficient of variation, R^2^, representing the fraction of the variance in expression that was attributable to the regression relationship. The regression using a sigmoidal function (modified tanh (×)) was highly significant at *p* = 0.014 (F test, *n* = 12). Error bars are standard errors of the mean from duplicate measurements. (**b**): The relationship between AMPK antisense inhibition construct (pPROF362) copy numbers and AMPK mRNA levels. The copy numbers of pPROF688 and pPROF362 in the DJ-1/AMPK double antisense cotransformants ranged from 7–704 and 38–307, respectively. The figure shows the coefficient of variation, R^2^, representing the fraction of the variance in expression that was attributable to the regression relationship. The regression using a sigmoidal function (modified tanh (x)) was highly significant at *p* = 3.7 × 10^−4^ (F test, *n* = 13). Error bars are standard errors from duplicate measurements. Each point represents the mean from 3 independent experiments for a pure, clonal culture of a single strain (cell line). Although the best fitting regression lines did not clearly show this, it is likely that the antisense inhibition effect approached a maximum at the highest copy numbers of the antisense inhibition constructs.

If the phenotypes caused by oxidative stress in DJ-1 knockdown strains are AMPK-mediated, they should be genetically suppressed by the simultaneous knockdown of AMPK expression. To determine if this was the case, cotransformants in which expression of both DJ-1 and AMPK had been antisense-inhibited were isolated and studied under oxidative stress conditions. The construct copy numbers in the resulting stable transfomants were determined using qPCR and their effect on expression of DJ-1 and the single AMPK catalytic subunit in *Dictyostelium* were measured using qRT-PCR as previously described [18]. Figure 2 confirms that in the double knockdown strains, the expression of DJ-1 (Figure 2a) and AMPK (Figure 2b) mRNAs were both reduced as expected, in a manner dependent on the copy number of the cognate construct.

### 3.3. The Combination of Oxidative Stress and Reduced Levels of DJ-1 Causes a Phototaxis Defect That Is AMPK-Mediated

**Figure 3:**
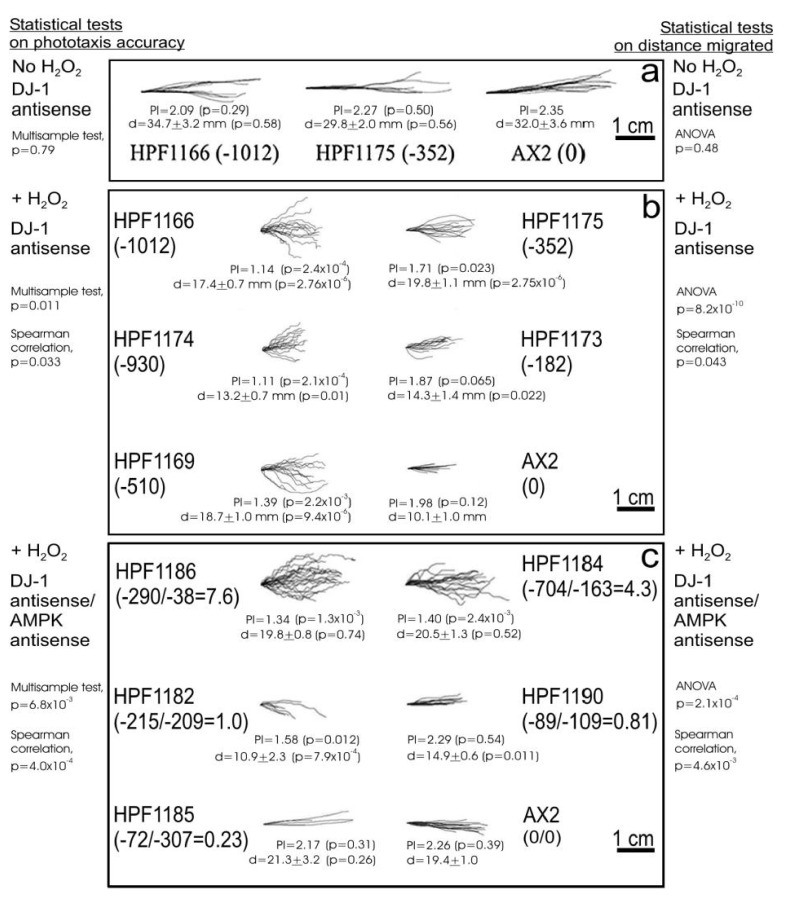
Phototaxis under oxidative stress by slugs of DJ-1 antisense transformants and DJ-1/AMPK double antisense cotransformants of the parental strain AX2. Slugs were formed from 2.5 × 10^6^ amoebae inoculated onto a 1 cm^2^ area in the center of each plate containing water agar supplemented with 900 µM H_2_O_2_ and allowed to migrate for 2 days with a lateral light source at 21 °C. The directions of travel were analyzed using directional statistics based on the von Mises circular normal distribution. PI, the phototaxis index, is the log of κ, the concentration parameter of the von Mises distribution (equivalent to the inverse of the variance of the directions). The mean distance migrated with standard errors is indicated for each set of trails. Two-sample and multisample tests of differences in phototactic accuracy were as described previously [37]. Two-sample and multisample tests of differences in the mean distance migrated used the *t*-test and ANOVA, respectively. H_2_O_2_ had no significant effect on the accuracy of phototaxis by the wild type parental strain (*p* = 0.46), but did significantly reduce the distance migrated (ANOVA, *p* = 2.0 × 10^−6^). (**a**): Digitized trails of DJ-1 antisense and parental (AX2) strains in the absence of H_2_O_2_. The numbers in brackets represent the copy numbers of pPROF688, the DJ-1 antisense-inhibition construct. (**b**): Digitized slug trails of DJ-1 antisense and parental (AX2) strains exposed to H_2_O_2_. The numbers in brackets represent the copy numbers of pPROF688, the DJ-1 antisense-inhibition construct. (**c**): Digitized slug trails of DJ-1/AMPK double antisense cotransformants and the parental strain (AX2) exposed to H_2_O_2_. The numbers in brackets represent the ratio between the copy numbers of pPROF688 (DJ-1 antisense-inhibition construct, numerator) and pPROF362 (AMPK antisense-inhibition construct, denominator). In all panels the light source is to the right of the figure. Phototaxis is unimpaired in the wild type AX2 with slug trails heading directly toward the light source. DJ-1 antisense-inhibited transformants display reduced accuracies of phototaxis in the presence of H_2_O_2_ and the severity correlates with increased copy numbers. The phototaxis defect of the DJ-1 antisense strains under oxidative stress was rescued by antisense-inhibition of AMPK in the cotransformants. The degree of rescue was dependent on the number of copies of each construct. When the ratio between pPROF688 (DJ-1 antisense-inhibition construct) and pPROF362 (AMPK antisense-inhibition construct) was less than or close to 1, the accuracy of phototaxis resembled that of the wild type AX2. When the ratio exceeded 1, the reduction in AMPK expression was not sufficient to overcome the effect of the reduction in DJ-1 expression. DJ-1 knockdown also increased the distance migrated (trail lengths) by the slugs in the presence of H_2_O_2_ (**b**), an effect that was also reversed by AMPK knockdown (**c**). This effect could be a result of differences in the aggregate/slug size formed by these strains (see later section on fruiting body morphology). Larger slugs are known to migrate faster and further than small slugs [44].

One of the aberrant phenotypes characteristic of mitochondrially diseased *Dictyostelium* strains is impaired phototaxis in the multicellular slug stage of the life cycle [24,30]. The impairment of phototaxis in mitochondrial disease arises because the compromised ability of the mitochondria to synthesize ATP results in chronic activation of the energy-sensing protein kinase AMPK [30]. Chronic AMPK hyperactivity in its turn dysregulates the signal transduction pathways that control phototaxis and other cellular activities. Since oxidative stress impairs mitochondrial respiratory function, it might be expected that a downstream phenotypic consequence of this would be a phototaxis defect. When we tested the effect of oxidative stress on wild type, parental AX2 slugs, we found that in fact there was no significant defect in phototaxis; therefore, the slugs migrated with their normal highly accurate orientation toward a lateral light source (Figure 3a, AX2).

Could the failure of oxidative stress to cause a phototaxis defect be due to a protective role for DJ-1? We previously showed that in the absence of oxidative stress, antisense inhibition of DJ-1 expression does not impair phototaxis [18] (Supplementary Figure S3). However, since DJ-1 is posttranslationally modified by oxidation at C117 under conditions of oxidative stress, it could play a protective role not evident in unstressed cells. To determine this, we examined whether the combination of oxidative stress and reduced expression of DJ-1 would cause phototaxis defects. DJ-1 knockdown alone had no effect on the accuracy of phototaxis or the distance migrated by the slugs (Figure 3a). However, Figure 3b shows that the accuracy of phototaxis was reduced in the DJ-1 knockdown strains in the presence of 900 µM H_2_O_2_ and that the severity of the defect was correlated with the number of copies of the DJ-1 antisense-inhibition construct (pPROF688) copy number. This result reveals a role for DJ-1 in protecting cells from the downstream consequences of mitochondrial dysfunction under oxidative stress conditions.

As the defect in phototaxis in mitochondrially diseased *Dictyostelium* strains is caused by chronic activation of the energy stress-sensing kinase AMPK [30], this could also be the case when oxidative stress is combined with a loss of the protection afforded by DJ-1. When we tested cotransformants in which both DJ-1 and AMPK were antisense-inhibited (Figure 3c), we found that the defects in phototaxis caused by the combined effects of oxidative stress and DJ-1 loss were suppressed by the knockdown of AMPK expression in the same cells. The extent of rescue of the defective phototaxis was dependent on the ratio of the copy numbers for the two antisense constructs—the greater the loss of DJ-1 expression is (caused by more copies of the DJ-1 antisense construct), the greater the extent of AMPK knockdown (copies of the AMPK antisense construct) that is required to counteract this loss. This result suggests that DJ-1 protects cells from the AMPK-mediated consequences of mitochondrial dysfunction arising from oxidative stress. When this DJ-1-mediated protection is reduced (or lost), AMPK hyperactivity under oxidative stress conditions produces a phototaxis defect.

### 3.4. Oxidative Stress Causes an AMPK-Dependent Exacerbation of the Phagocytosis Defect Produced by DJ-1 Loss

**Figure 4:**
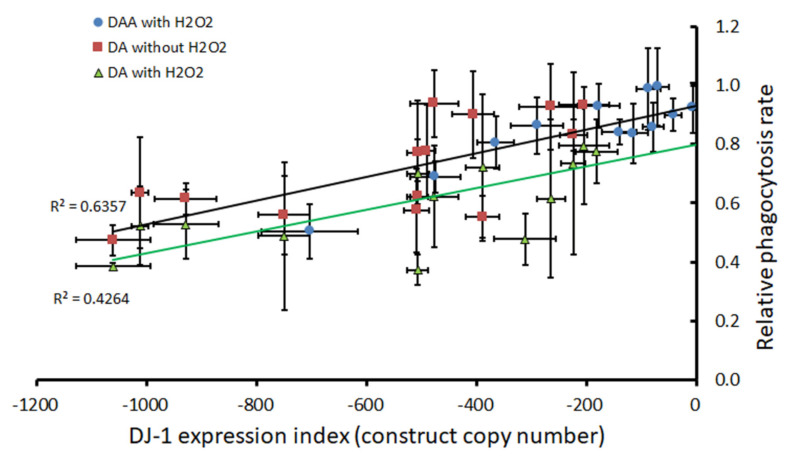
The effect of oxidative stress, DJ-1 and AMPK knockdown on phagocytosis. Phagocytosis rates of DJ-1 antisense (DA) and DJ-1/AMPK double antisense (DAA) cotransformants exposed to H_2_O_2_, compared to DJ-1 antisense transformants without exposure to H_2_O_2_. The phagocytosis rates were measured as the rate of uptake in shaken suspension of DS-Red-expressing (fluorescent) *E coli* cells (bacteria taken up per amoeba per hour), normalized to those measured in the same experiment for the parental strain AX2. These were plotted against the DJ-1 expression index measured as per the previously established convention of using the negative of the antisense inhibition construct (pPROF688) copy numbers [18]. Error bars are standard errors of the mean from 3 independent experiments. The results for DJ-1 knockdown (DA) without H_2_O_2_ were previously described [18] (Supplementary Figure S4). R2 represents the fraction of the variance in expression that was attributable to the regression relationship. Multiple regression analysis was performed to determine the significance of regression parameters and lines were fitted by linear least squares to pass through the intercepts derived therefrom (Supplementary Table S1).

We previously reported that DJ-1 knockdown in *Dictyostelium* impairs phagocytosis [18]. Here, we examined whether this phenotype was made worse by oxidative stress, and whether this exacerbation was also dependent on AMPK. Phagocytosis rates decreased with a decreasing expression of DJ-1, with or without exposure to 450 µM H_2_O_2_. Furthermore, multiple regression analysis showed that the H_2_O_2_ exposure caused an additional, constant reduction in the rate of phagocytosis in the wild type and the DJ-1 antisense strains, regardless of the DJ-1 expression levels (Figure 4, Supplementary Table S1 and Figure S4). This suggests that oxidative stress impairs phagocytosis in a manner that is additional to that mediated by DJ-1 loss.

Does this additional phagocytosis-impairing pathway also require AMPK activity? The effect of antisense-inhibiting both AMPK and DJ-1 expression in the same cells showed that it does. Thus, AMPK knockdown rescued cells from the additional effect of H_2_O_2_ on phagocytosis rates, returning them to the level expected for unstressed cells with the same level of DJ-1 expression (Figure 4). It was previously shown that phagocytosis is unaffected by mitochondrial dysfunction and AMPK activity in otherwise unstressed cells [30]. The present result shows that in oxidatively stressed cells, phagocytosis becomes sensitive to and inhibited by AMPK signaling. The simplest explanation is that the oxidized form of DJ-1, present only in oxidatively stressed cells, is inhibited by AMPK, which is activated both by oxidative stress and mitochondrial dysfunction [30,45]. Phagocytosis would be affected by AMPK only under conditions of oxidative stress, because only then is the oxidized form of DJ-1 available for inhibition by AMPK.

### 3.5. Pinocytosis Is Not Affected Significantly by DJ-1 Knockdown under Conditions of Oxidative Stress

**Figure 5:**
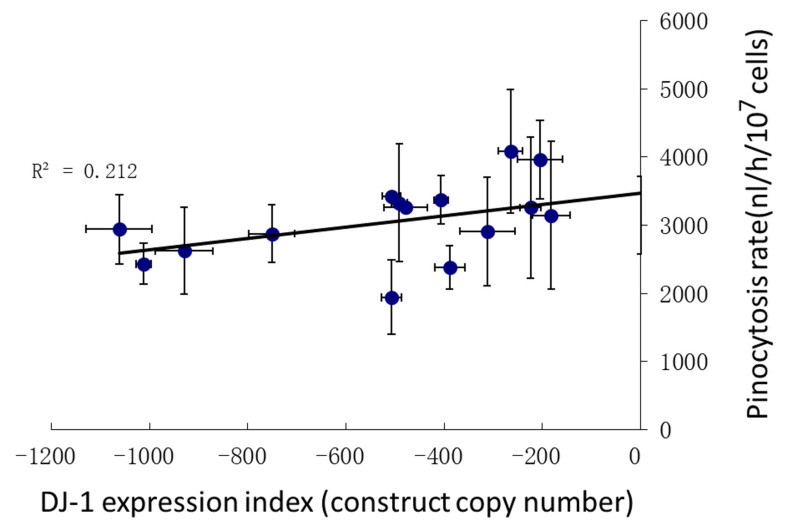
Pinocytosis by DJ-1 antisense transformants under oxidative stress. The pinocytosis rates of the DJ-1 antisense transformants showed a slight decrease as the copy numbers of DJ-1 antisense-inhibition construct (pPROF688) increased compared with AX2, but this was not significant (*p* = 0.073) (F test, *n* = 16). R^2^ represents the fraction of the variance that was attributable to the regression relationship. Error bars are standard errors of the mean from 3 independent experiments.

The foregoing results showed that oxidative stress inhibits mitochondrial respiration and impairs phagocytosis in an AMPK-dependent manner, worsening the defect in phagocytosis caused by DJ-1 loss. An alternative means by which *Dictyostelium* can acquire nutrients is by macropinocytosis, a process that exhibits both similarities and differences with phagocytosis at the phenomenological and molecular levels [46]. We previously showed that DJ-1 expression levels have only a very slight effect on pinocytosis, such that the rates of pinocytosis were only about 20% lower in unstressed cells with more than 1000 copies of the DJ-1 antisense construct [18] (Supplementary Figure S5). In view of the significant impact of oxidative stress on phagocytosis, we determined whether exposure to H_2_O_2_ would have similar effects on pinocytosis. Pinocytosis was assayed in AX2 and DJ-1 antisense transformants exposed to 450 μM H_2_O_2_. The rate of pinocytosis in oxidatively stressed cells did appear to be slightly reduced by DJ-1 knockdown, with the regression indicating a 30%reduction at copy number 1000. However, in this case the effect did not reach statistical significance (Figure 5), perhaps because in the previously reported work a greater expression range for DJ-1 was tested by also including DJ-1 overexpression strains [18] (Supplementary Figure S5). We conclude that oxidative stress has little impact on the slight reduction in pinocytosis caused by DJ-1 knockdown.

### 3.6. DJ-1 Protects Cells from AMPK-Dependent Inhibition of Growth Caused by Oxidative Stress

In *Dictyostelium* cells with defects in mitochondrial function, AMPK impairs cell growth and division without affecting nutrient uptake by phagocytosis or pinocytosis [30]. Although DJ-1 knockdown has large effects on phagocytosis that are exacerbated significantly by oxidative stress, this is not the case for pinocytosis, as shown by the results in the preceding section. By measuring proliferation in liquid medium, we were, therefore, able to investigate the growth-regulating interactions between DJ-1, AMPK and oxidative stress without the superimposed complications of large effects on nutrient uptake.

**Figure 6:**
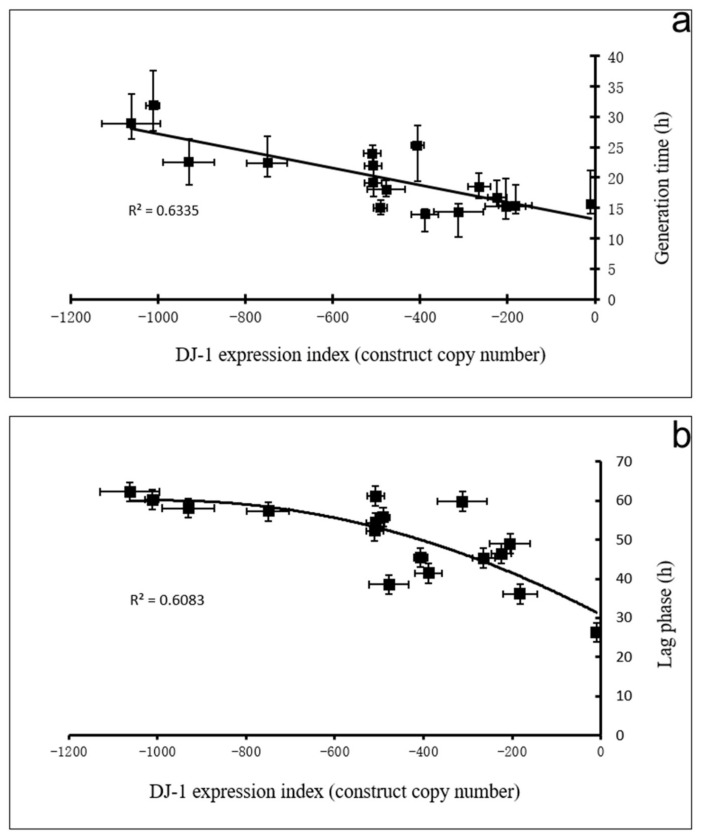
DJ-1 knockdown dramatically inhibits growth in oxidatively stressed cells. (**a**): The generation time of DA transformants grown in liquid media under oxidative stress (450 μM H_2_O_2_) increased with increasing DJ-1 antisense inhibition copy numbers. R^2^ represents the fraction of the variance that was attributable to the regression relationship. The regression was highly significant at *p* = 1.8 × 10^−4^ (F test, *n* = 16). Error bars for the generation times indicate averages for the 95% confidence limits from 3 independent experiments. Error bars for the copy numbers are standard errors from 3 independent experiments. (**b**): The lag time of DA transformants under oxidative stress increases (450 μM H_2_O_2_) with increasing DJ-1 antisense inhibition copy number. R^2^ represents the fraction of the variance that was attributable to the regression relationship. The regression was highly significant at *p* = 1.7 × 10^−4^ (F test, *n* = 16). Error bars are standard errors from 3 independent experiments. Lines were fitted by least squares. R^2^ indicates the fraction of the variance explained by the regression.

**Figure 7:**
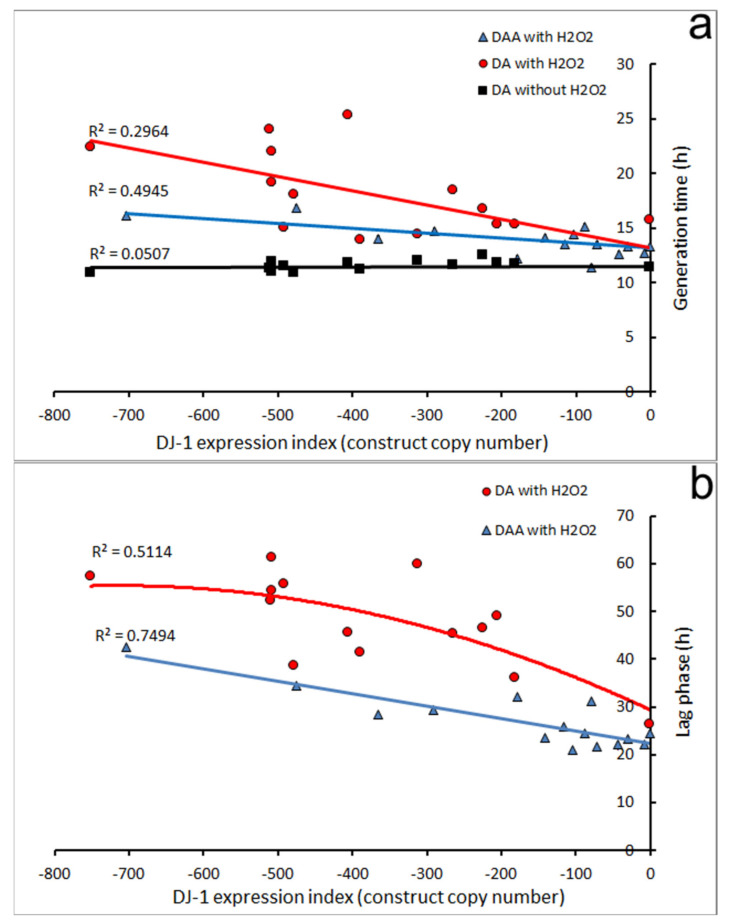
The effect of oxidative stress, DJ-1 and AMPK knockdown on growth in liquid. (**a**): The comparison of generation time between DJ-1 antisense (DA) strains with or without H_2_O_2_ and DJ-1/AMPK double antisense) DAA cotransformants with H_2_O_2_. The multiple regression analysis of generation time for DA and DAA strains is shown in Supplementary Table S2. The error bars are not plotted because they made the plot too cluttered. To allow for the easy comparison of results in the presence of H_2_O_2_ with those obtained without H_2_O_2_, the previously reported results without H_2_O_2_ [16] are included here. In the absence of the DJ-1 knockdown construct (copy number 0), the H_2_O_2_ caused the generation time to increase significantly (*p* = 0.039) by 1.7 h whether or not the AMPK antisense construct was present (intercepts). In the absence of H_2_O_2_, the knockdown of DJ-1 had no significant effect on the generation time (black line), but in the presence of H_2_O_2,_ growth was dramatically slowed by DJ-1 knockdown in a copy-number-dependent manner (slope = −0.013, *p* = 9 × 10^−9^, red line). When AMPK expression was also knocked down (blue line), the effect of DJ-1 loss was still significant (*p* = 5.9 × 10^−4^), but was reduced 3-fold (slope = −0.0045). This shows that in the presence of H_2_O_2_, the impairment of growth caused by DJ-1 loss is largely mediated by AMPK. (**b**): The comparison of lag time between DJ-1 antisense (DA) and DJ-1/AMPK double antisense (DAA) strains exposed to H_2_O_2_. In the absence of H_2_O_2_, the lag time was 0 in all strains, because they were already growing in exponential phase at the start of the experiment. In the presence of H_2_O_2_, the DJ-1 knockdown caused a dramatic copy number-dependent increase in the lag time. This longer lag time was reduced significantly when AMPK expression was also knocked down, showing that the increased lag time caused by DJ-1 knockdown is mediated by AMPK. The multiple regression analysis of lag times for DJ-1 antisense (DA) and DJ-1/AMPK double antisense (DAA) strains is shown in Supplementary Table S3. The error bars are not plotted because they made the plot too cluttered. Lines were fitted by linear least squares to pass through the intercepts determined by the multiple regressions in Supplementary Tables S2 and S3. R^2^ indicates the fraction of the variance explained by the regression.

We had previously observed that changing DJ-1 expression levels had only very slight effects on proliferation in liquid medium; therefore, the generation times were extended by only about 14% in unstressed cells with more than 1000 copies of the DJ-1 antisense construct [18] (Supplementary Figure S5). We, therefore, first determined whether DJ-1 knockdown had more dramatic effects on growth in liquid medium in the presence of oxidative stress (Figure 6). The results showed that in oxidatively stressed cells, knocking down DJ-1 had a dramatic copy number-dependent effect both on the proliferation rate (generation time, Figure 6a) and the lag phase (Figure 6b). As these experiments use cells already growing in logarithmic phase in the test medium (HL-5), there is no lag phase in cells not exposed to H_2_O_2_. However, even in wild type cells (0 copies of the DJ-1 antisense construct), the presence of H_2_O_2_ was sufficient to cause a lag time of about 30 h before growth begins (intercept, Figure 6b). Thereafter the cells grow more slowly than would otherwise be the case (ca. 13 h generation time compared to 11 h without H_2_O_2_, intercepts Figure 6a and Figure 7a, Supplementary Figure S5a). The knocking down expression of DJ-1 under oxidative stress conditions dramatically extended the lag time and the generation time in a copy number-dependent fashion. This suggests that DJ-1 protects against the effect of H_2_O_2_ on growth in liquid medium; therefore, its loss exacerbates the adverse effects of oxidative stress.

To determine whether this impaired growth under oxidative stress is caused by the activation of AMPK, the cotransformants containing both the DJ-1 and the AMPK antisense constructs (DAA) were also tested. The effect of DJ-1 knockdown on both the lag time and generation time in oxidatively stressed cells was substantially (but incompletely) rescued in the DJ-1/AMPK double antisense cotransformants (Figure 7, with corresponding multiple regressions in Supplementary Tables S2 and S3). The generation times and lag times of the cotransformants were dramatically reduced compared to what they would have been without the AMPK antisense construct in the same cells.

The AMPK knockdown also appeared to relieve slightly the effect of H_2_O_2_ alone (i.e., no DJ-1 knockdown) on growth in liquid, but this effect (compare the blue and red line intercepts–0 copies of the DJ-1 knockdown construct) was not statistically significant in multiple regression analysis (Supplementary Tables S2 and S3). However, the slopes of the regressions (blue and red lines) relating the DJ-1 antisense construct copy number to generation times and lag times in the presence of H_2_O_2_ were significantly altered by knocking down AMPK. Thus, the dependence of growth in liquid on the DJ-1 expression level is itself altered (line slopes changed), with the impairment caused by oxidative stress in DJ-1 knockdown strains being largely relieved by AMPK knockdown. These results show that in oxidatively stressed cells the dramatic effect of DJ-1 loss on growth in liquid is mediated largely by AMPK. The simplest explanation is that (as in other organisms) oxidative stress activates AMPK and the activated AMPK inhibits growth (as previously shown [30]). However, at the same time in wild type cells, DJ-1 becomes oxidized, and the oxidized form prevents the growth-inhibiting effects of AMPK. This protection from AMPK’s growth-inhibiting activity under oxidative stress conditions is reduced by DJ-1 knockdown.

### 3.7. DJ-1, AMPK and Oxidative Stress Interact in Regulating Fruiting Body Morphology

**Figure 8:**
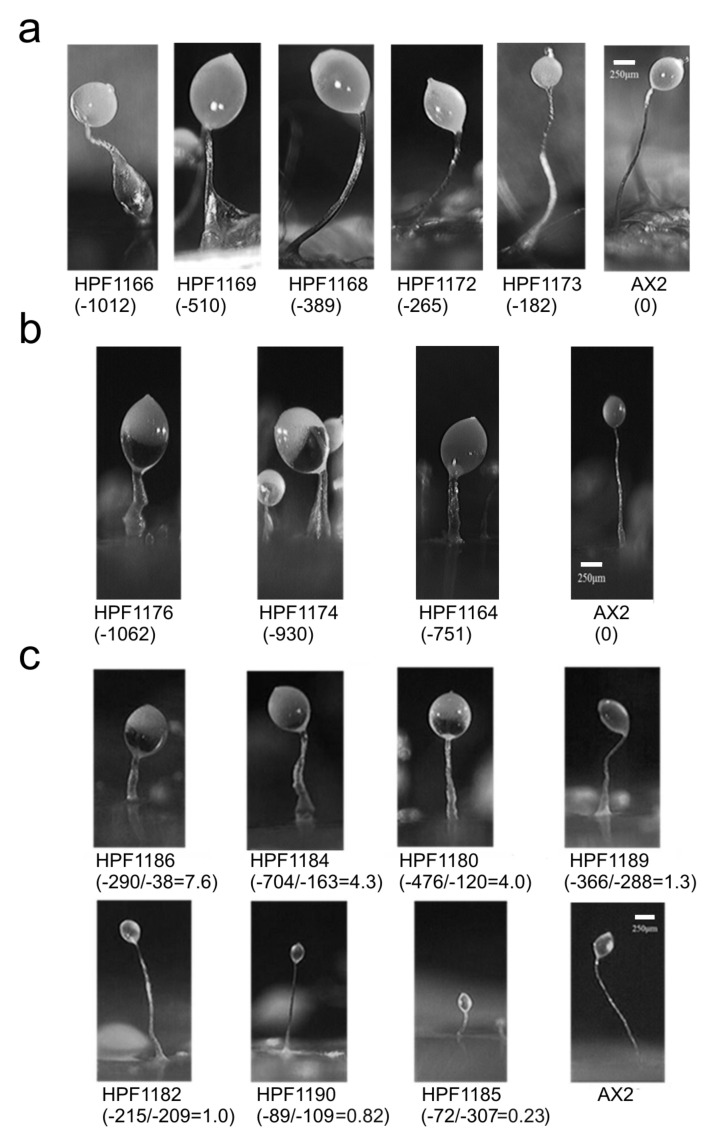
The morphology of the DJ-1 antisense and DJ-1/AMPK double antisense strains under oxidative stress. (**a**): The fruiting body morphology of DJ-1 antisense transformants on KK2 plates containing 900 μM H_2_O_2_. The numbers in brackets are the copy numbers of the DJ-1 antisense-inhibition construct (pPROF688). Fruiting bodies were larger at higher copy numbers. (**b**): The fruiting body morphology of DJ-1 antisense transformants on SM plates containing 900 μM H_2_O_2_. The numbers in brackets are the copy numbers of the DJ-1 antisense-inhibition construct (pPROF688). Fruiting bodies were larger at higher copy numbers. (**c**): The fruiting body morphology of DJ-1/AMPK double antisense cotransformants on SM plates containing 900 μM H_2_O_2_. The numbers in brackets are the copy numbers of DJ-1 antisense construct (pPROF688) and AMPK antisense construct (pPROF362) and their ratio. As the copy numbers of pPROF688 in DA transformants increased, the fruiting bodies became more defective, with larger sori and thicker, shorter stalks (shown in **a**,**b**). This defect could be rescued in the DAA cotransformants if the ratio of the DJ-1 antisense construct (pPROF688) and the AMPK antisense construct (pPROF362) copy numbers was close to or less than 1. If the ratio was greater than 1, then the amount of AMPK antisense inhibition was not sufficient to restore the defect. At ratios significantly below 1.0, the fruiting bodies became smaller, but appeared normally proportioned. All photographs were taken and all images are presented at the same magnification. All images were taken at the same magnification. Scale bars represent 250 µm.

The fruiting body morphology of the DJ-1 antisense (DA) transformants in the absence of H_2_O_2_ was defective with larger sori and thicker, shorter stalks [18] (Supplementary Figure S6). In the presence of H_2_O_2_, DJ-1 knockdown produced fewer fruiting bodies, again with thicker and shorter stalks (Figure 8a,b). The severity of this defect in the presence of H_2_O_2_ was dependent on the DJ-1 antisense-inhibition construct (pPROF688) copy number and, for a given copy number, appeared to be more severe than in the absence of H_2_O_2_ [18] (Supplementary Figure S6). However, the morphology of the wild type strain AX2 appeared little affected by the oxidative stress alone. These results support the idea of a protective role of DJ-1. The morphological defects resembled those in mitochondrially diseased and AMPK overexpression strains [30] and are consistent with a role for AMPK in regulating the number of aggregation centers formed in the early stages of morphogenesis [47]. To determine if the morphological defect in the DJ-1 antisense transformants was mediated by AMPK, the DJ-1/AMPK double antisense cotransformants were analyzed. These cotransformants showed varied morphologies that depended on the ratio between the copy numbers of the DJ-1 antisense-inhibition construct (pPROF688) and the AMPK antisense-inhibition construct (pPROF362) (Figure 8c). The morphological defect worsened with greater numbers of copies of the DJ-1 antisense construct, but this was reversed by equivalent increases in the number of copies of the AMPK antisense construct. Thus, the large sori and short, thick stalks of DJ-1 antisense transformants do appear to be caused by the activation of AMPK.

## 4. Discussion

The loss of the function of DJ-1 has been found to be associated with autosomal recessive, early onset PD in mammalian cells by functioning as a molecular chaperone, antioxidant, transcriptional regulator and/or protease [2,4,7,8,11,12]. Mitochondrial dysfunction has been implicated in the pathogenesis of PD and DJ-1 appears to protect the cells from the downstream consequences of mitochondrial dysfunction, but the mechanism is not clear [15]. As an established mitochondrial disease model, *D. discoideum* offers the opportunity to determine if the loss or reduction in DJ-1 activity causes mitochondrial dysfunction and AMPK-mediated, aberrant phenotypes in the presence or absence of oxidative stress. In *Dictyostelium*, defects in mitochondrial respiration produce reliable phenotypes that, except for some isolated Complex I-specific defects, are consistent, regardless of the underlying mitochondrial defect [22,24]. If the loss of DJ-1 causes mitochondrial respiratory dysfunction, the antisense inhibition of DJ-1 expression would impair respiration and phenocopy the well-established outcomes of mitochondrial disease, and these phenotypes would be rescued by the antisense inhibition of AMPK. However, we reported that this is not the case for unstressed *Dictyostelium* cells, for which we found that the loss of DJ-1 stimulates mitochondrial respiration and does not phenocopy mitochondrial dysfunction [18]. Mitochondrial dysfunction in *Dictyostelium* typically impairs growth without affecting endocytosis in the unicellular stage of the life cycle and impairs phototaxis in the multicellular slug stage. By contrast, the loss of DJ-1 impairs endocytosis, particularly phagocytosis, and has no significant effect on phototaxis.

**Figure 9:**
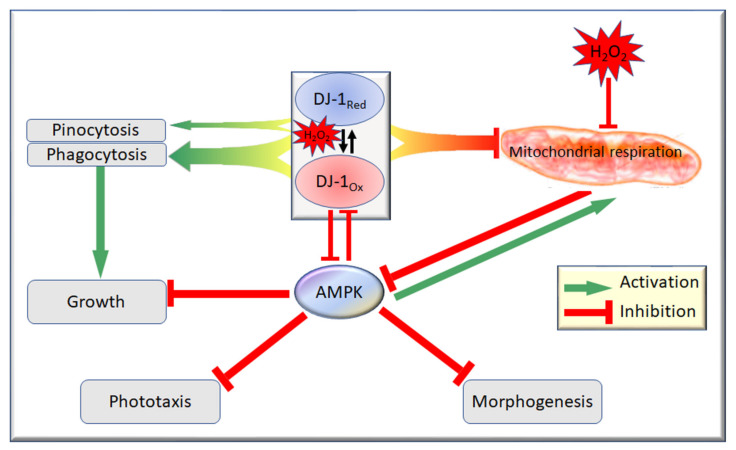
Model for phenotypic consequences of interactions between DJ-1, AMPK, mitochondrial respiration and oxidative stress. Mitochondrial respiration is inhibited separately by oxidative stress and DJ-1, both in its oxidized (DJ-1_Ox_) and reduced (DJ-1_Red_) states (indicated by the bifurcated base of the arrow). DJ-1 also activates phagocytosis (broad arrow) and, to a slight extent, (narrower arrow) pinocytosis, regardless of its oxidation state (indicated by the bifurcated base of the arrows). DJ-1_Ox_, but not DJ-1_Red_, inhibits and is inhibited by AMPK. Under oxidative stress, mitochondrial respiration is inhibited, ATP production is compromised and AMPK is activated. At the same time, DJ-1 is oxidized and inhibits AMPK, opposing the effects of impaired respiration and protecting cells from the downstream phenotypic consequences of chronic AMPK hyperactivity.

However, various studies have shown that DJ-1 protects cells from the oxidative stress caused by mitochondrial inhibitors or oxidizing agents, including rotenone, MPP+, paraquat, 6-hydroxydopamine (6-OHDA) or H_2_O_2_ that impair mitochondrial function [8,48,49,50,51]. This raises the possibility that under oxidative stress conditions, DJ-1 might play an additional role in protecting the mitochondria from oxidative damage. We showed, here, that H_2_O_2_ impairs mitochondrial respiration in *Dictyostelium* and that it does not alter the enhancement of the mitochondrial respiration caused by DJ-1 loss. However, the loss of DJ-1 did render the cells more sensitive to the downstream phenotypic consequences of exposure to this strong oxidant. The simplest way to draw together the results supporting this conclusion is in the form of a model for multiple interactions between oxidative stress, the mitochondria, AMPK and DJ-1 (Figure 9).

The model’s key features, along with the corresponding experimental results and literature citations, may be summarized as follows:

1. Oxidative stress in the form of H_2_O_2_ acts in two places to (a) convert DJ-1 to its oxidized form at the conserved, oxidizable cysteine C117 [10,18] and (b) separately impair the mitochondrial respiratory function (Figure 1).
2. Regardless of its oxidation state, DJ-1 impairs, and its loss enhances, the mitochondrial respiratory function [18] (Figure 1). DJ-1 is found in the cytosol, both in unstressed and oxidatively stressed *Dictyostelium* cells; therefore, any effects it has on the mitochondria must be indirect [18].
3. Regardless of its oxidation state, DJ-1 is an activator of phagocytosis [18] (Figure 4, Supplementary Figure S4) and also slightly activates pinocytosis [18] (Figure 5, Supplementary Figure S5). This elicits corresponding effects on the growth on bacterial lawns and in liquid medium [18] (Figure 6 and Figure 7; Supplementary Figures S4 and S5).
4. AMPK is activated by the mitochondrial dysfunction and consequent reduction in ATP synthesis [24,30,50,51,52] caused by oxidative stress.
5. In its reduced state, DJ-1 has little or no interaction with AMPK. In unstressed cells most DJ-1 is in the reduced state; therefore, altering its expression exerts no detectable effect on phototaxis (Supplementary Figure S3) and relatively small effects on morphogenesis (Supplementary Figure S6) [18]. For the same reason, in the absence of oxidative stress, AMPK activation by mitochondrial disease has no detectable effect on phagocytosis or pinocytosis [30].
6. In its oxidized state, DJ-1 becomes sensitive to inhibition by AMPK. As a consequence of this, oxidative stress contributes to an AMPK-mediated impairment of phagocytosis (Figure 4), additional to that caused by DJ-1 loss. AMPK antisense-inhibition reverses the effect of oxidative stress on phagocytosis without significantly altering the inhibitory effect of DJ-1 knockdown (Figure 4). The same probably occurs in relation to pinocytosis, which was already shown to be slightly inhibited by DJ-1 loss [18], although this could not be detected statistically in the present experiments (Figure 5). The magnitude of this AMPK-dependent effect is in proportion to the relative magnitude of DJ-1′s influence on these two types of endocytosis [18].
7. In its oxidized state, DJ-1 becomes capable of inhibiting AMPK. As a consequence of this, it opposes and limits the activation of AMPK caused by oxidative stress. For this reason, wild type DJ-1 at normal levels prevents oxidative stress from causing dramatic defects in phototaxis, morphogenesis or growth in liquid. However, the loss of DJ-1 allows for the unrestrained activation of AMPK by oxidative stress; therefore, the combination produces a dramatic AMPK-dependent impairment of growth (Figure 6 and Figure 7), phototaxis (Figure 3) and morphogenesis (Figure 8).

It should be noted that none of the results presented are able to demonstrate that the observed genetic interactions are a consequence of direct biochemical actions. In particular, the mutually inhibitory interactions between the oxidized form of DJ-1 and AMPK could be mediated by intermediary players. Similarly, the inhibitory effects of oxidative stress on mitochondrial respiration need not be direct or even a result of oxidative biochemical damage to cellular or mitochondrial components. Instead, the H_2_O_2_ could inhibit respiration via intracellular signaling pathways and molecules other than those considered here. Furthermore, there may be additional competing interactions that participate. For example, AMPK is known to be activated by oxidative stress via a mechanism that involves phosphorylation by the upstream kinase, Tak1, and is independent of the cellular energy status [45]. These possibilities should be a subject of future research.

In the model in Figure 9, the oxidation of DJ-1 exerts protective effects by changing some (mutual inhibitory interactions with AMPK signaling) but not all (activation of endocytosis, inhibition of respiration) of the protein’s functions. We have not directly shown that the oxidation of C117 is, indeed, responsible for the protective actions of DJ-1. However, in other systems, this has been shown. Canet-Aviles et al. [10] showed that the oxidation of human DJ-1 at the equivalent cysteine (C106) is required for the protective effects of DJ-1. Taira et al. [11] expressed DJ-1 in human neuroblastoma SH-SY5Y cells exposed to hydrogen peroxide and found that, when overexpressed, DJ-1 eliminated H_2_O_2_-induced cell death by auto-oxidizing at residue C106, whereas the knockdown of DJ-1 rendered the cells susceptible to a H_2_O_2_, MPP+ or 6-OHDA-induced cell death. This cysteine residue is located after strand β7, according to the crystal structure of human DJ-1 [53,54,55]. It is highly conserved among all of the members of the ThiJ/PfpI superfamily to which DJ-1 belongs and has been proposed to be a catalytic residue for intracellular proteases of the PfpI family [56]. Mitsumoto and Nakagawa [57] also showed that C106 in human DJ-1 is the most plausible residue responsible for the hydroperoxide-responsive pI (isoelectric point) shift of DJ-1 from 6.2 to 5.8 after oxidation, as this shift is consistent with formation of cysteine sulfinic acid at residue C106. It is, therefore, likely that in *Dictyostelium* C117, oxidation mediates the protective effects of DJ-1 in oxidatively stressed cells, as C106 does in human DJ-1 [10,14]. Nevertheless, this should be explicitly tested in future work by mutating C117 to prevent or to constitutively mimic its oxidation.

In otherwise unstressed cells, we found that DJ-1 knockdown caused an elevation of mitochondrial respiration [18] (Figure 1). Similar mitochondrial hyperactivity was recently reported in *Dictyostelium* cells overexpressing the wild type or PD-associated mutant forms of α-synuclein [58], in cultured human neuroblastoma cells treated with exogeneous α-synuclein [59], in lymphoblasts from idiopathic PD patients [28] and in fibroblasts from genetic PD patients with Parkin mutations [29]. This raises the intriguing possibility that mitochondrial hyperactivity is an initiating event in these different types of PD. Mitochondrial hyperactivity can as easily cause elevated levels of reactive oxygen species (ROS) and oxidative stress as a mitochondrial electron transport blockade does [28]. It is possible that where DJ-1 activity has been compromised by a mutation or other causes, the resulting mitochondrial hyperactivity could be an initiating event that produces oxidative stress and downstream cytopathology, including the AMPK-dependent defects reported here for *Dictyostelium*, or the AMPK-dependent cell death reported by others [45]. Since one of AMPK’s well-established actions is to activate mitochondrial biogenesis and respiratory function, the outcome could be a vicious cycle leading to the chronic elevation of both AMPK activity and oxidative stress. In long-lived, metabolically highly active neuronal cells such as those in the substantia nigra, the resulting accumulation of oxidative damage could ultimately produce mitochondrial defects and cell death [28].

The evident protective role played by DJ-1 in *D. discoideum* supports the protective function of DJ-1 indicated by multiple cell lines and animal models [2,11,12,13,60]. However, these models measured the cytopathological consequences of oxidative stress, without directly measuring mitochondrial function. Our results suggest an alternative interpretation, namely that DJ-1′s protective role is not exerted in the mitochondria, nor is it directed at mitochondrial respiratory function. Instead it is exerted in the cytosol and directed primarily at downstream cytopathological events, including at least some that are AMPK-dependent.

## Data Availability

This work did not involve the creation of large data sets. The raw data is available on request.

## Supplementary Materials

The following are available online at https://www.mdpi.com/article/10.3390/cells10081874/s1: Supplementary Methods: Western blotting; Figure S1: Seahorse respirometry assay of mitochondrial function in intact *Dictyostelium* cells. Figure S2: Confirmation that DJ-1 C117 is oxidized in *D. discoideum* under conditions of oxidative stress. Figure S3: Phototaxis by slugs of *D. discoideum* DJ-1 transformants with altered DJ-1 expression. Figure S4: DJ-1 upregulates growth on bacterial lawns and phagocytosis. Figure S5: DJ-1 modestly upregulates growth in liquid medium and pinocytosis. Figure S6: DJ-1 knockdown causes aberrant *Dictyostelium* morphogenesis. Table S1: Multiple regression analysis of effects on phagocytosis rates of DJ-1 knock down, DJ-1/AMPK double knock down and oxidative stress. Table S2: Multiple regression analysis of generation time for DJ-1 antisense and DJ-1/AMPK double antisense transformants in the presence of H_2_O_2_. Table S3: Multiple regression analysis of lag time for DJ-1 antisense and DJ-1/AMPK double antisense transformants in the presence of H_2_O_2_.
