# Supplementary material for: The Parkinson’s Disease-Associated Protein DJ-1 Protects Dictyostelium Cells from AMPK-Dependent Outcomes of Oxidative Stress
Source: Cells. 2021 Jul 23;10(8):1874. doi: 10.3390/cells10081874 (PMC8392454; doi:10.3390/cells10081874)
Supplement: Supplementary file 1 [file cells-10-01874-s001.zip › cells-1194071-supplementary.pdf]

Supplementary Information

Supplementary methods

Western blotting

A crude protein lysate was prepared by lysing 5 x 10^5 growth phase amoebae on ice in 15 μL Laemmlii buffer containing 1 μl 25X proteinase inhibitor cocktail (Roche). The sample was then boiled for 10 min before loading 300 μg (Bradford assay) of *D. discoideum* protein onto a 12 % SDS-PAGE gel for gel electrophoresis. The mini Trans-Blot Turbo™ blotting apparatus (Bio-Rad) was used for western transfer to mini PVDF membrane as recommended by the manufacturer's manual. To detect the DJ-1 protein by Enhanced Chemifluorescence, a rabbit anti-human-DJ-1 monoclonal antibody (1:700, Anti-PARK7/DJ1 antibody [MJF-R16 (66-5)], Abcam) was used in conjunction with fluorescein-tagged anti-rabbit-IgG secondary antibody and an anti-fluorescein alkaline-phosphatease-conjugated Fab fragment (GE Healthcare Amersham). The antibody was raised against a synthetic peptide corresponding to the oxidized form of the C-terminal half of the human DJ-1 protein. Other commercial antibody preparations described as specific for this oxidized cysteine failed to detect any bands in any samples from wild type or DJ-1 overexpressing strains, regardless of whether they had been exposed to oxidative stress. There are no other oxidizable cysteine residues in this sequence or in the corresponding *Dictyostelium* sequence. The result was scanned on a Storm 860™ Phosphorimager.

Supplementary results

Supplementary Figure S1.

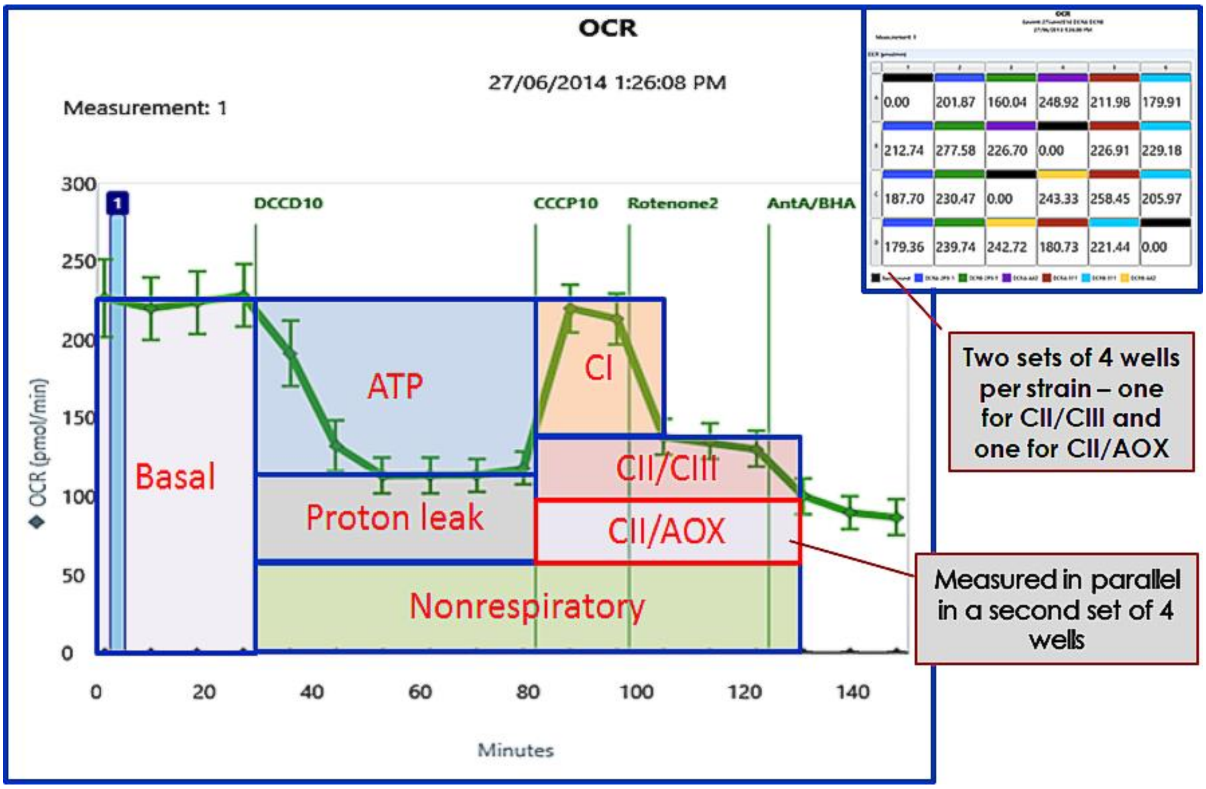

Supplemental Figure S1. Seahorse respirometry assay of mitochondrial function in intact *Dictyostelium* cells. The assay was described previously[1]. Respiratory electron transport in *Dictyostelium* is similar to that in mammalian mitochondria except that *Dictyostelium* still possesses an ancestral Alternative Oxidase (AOX) which can receive electrons from either Complex I or II via ubiquinone and pass them to molecular O_2. It thus provides an alternative to Complex III and IV for the passage of electrons to O_2.

The main part of the Figure shows an example real time trace of the O_2 Consumption Rate (OCR) by intact *Dictyostelium* cells plated at a density of 1 x 10^5 cells per well into a 24 well assay plate in the pattern shown in the inset. For a given strain and set of conditions two sets of 4 wells are inoculated, indicated by the colour coding of the wells eg. the blue and green well sets could be used for a particular strain treated with H_2O_2 and the red and cyan sets for the same strain without H_2O_2 treatment. The trace shown is for the set of 4 green-coded wells. The central pairs of yellow- and purple-coded wells always included the control strain AX2 under standard assay conditions (no H_2O_2). This is always used as an internal control for the quality of the assay, since the behaviour of this strain in this assay is thoroughly known. The black-coded wells are for measurement of background signal and they contain no cells, but all of the same buffers, ingredients and additions during the assay.

The assay is set up so that at the indicated times, specific inhibitors are added to the wells. After measurement of basal respiration, the O_2 Consumption Rate (OCR) was measured after successive additions of ATP synthase inhibitor oligomycin, the proton ionophore CCCP (to uncouple respiration, allowing maximum O_2 consumption), the Complex I inhibitor rotenone and either the Complex III inhibitor antimycin A (in one set of 4 wells per paired sets) or the Alternative Oxidase (AOX) inhibitor BHAM (in the other set of 4 wells per paired sets). After the assay, the calculated contributions of Complex II coupled to Complex III (measured by the decreased OCR after antimycin addition) and Complex II coupled to AOX (measured by the decreased OCR after BHAM addition) are added together to determine total Complex II activity. The “nonrespiratory” or “nonmitochondrial” component of respiration is the OCR obtained as the Maximum OCR after CCCP addition minus the contributions of Complex I and Complex II. The other components of respiration are calculated as indicated in the Figure eg. the “proton leak” is the difference between the OCR after oligomycin addition and the calculated “nonrespiratory” OCR. The error bars in the real time OCR trace represent standard errors of the mean for the 4 wells per set. They reflect technical variation within the experiment.

Supplementary Figure S2.

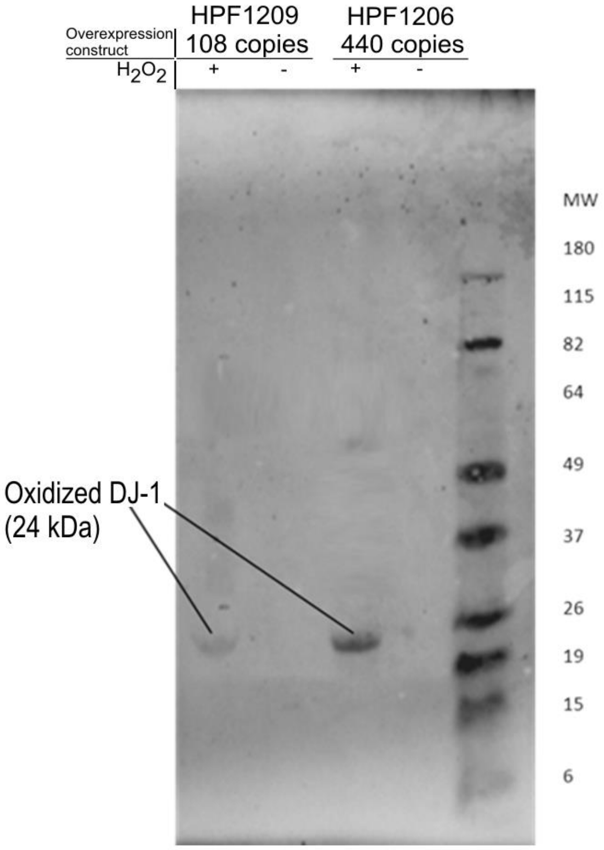

Supplementary Figure S2. Preliminary confirmation that DJ-1 C117 is oxidized in *D. discoideum* under conditions of oxidative stress. Two DJ-1 overexpressing *D. discoideum* strains (HPF1206 and HPF1209) were grown in HL-5 at 21 C shaking. The same strains were also grown in HL-5 containing 450 µM H_2O_2. The amoebae were harvested, lysed and the protein extract run on SDS-PAGE and western blotted. The oxidized DJ-1 protein was detected in *D. discoideum* strains exposed to H_2O_2, more of it in the strain with the higher number of copies of the overexpression construct. The amount of protein loaded per well was 300 µg (measured separately for each sample using the Bradford assay). To determine if C117 is oxidized in *D. discoideum* DJ-1, a monoclonal antibody which very specifically detects the oxidized form of C106 in human DJ-1 was used (Anti-PARK7/DJ1 antibody [MJF-R16 (66-5)], Abcam). This antibody failed to detect any protein in the parental strain AX2, with or without exposure to oxidative stress (not shown), because the level of expression of DJ-1 from the endogeneous gene was below the limits of detection in western blots. However it did detect a single band of the expected size in two different DJ-1 overexpression strains, only after exposure to an oxidative stress (450 µM H_2O_2 for 24 hrs). As expected, the band was more intense in the strain with the higher number of copies of the overexpression construct. These results provide preliminary confirmation that C117, the *D. discoideum* equivalent of human C106, is oxidized as a result of oxidative stress like its human counterpart.

Supplementary Figure S3.

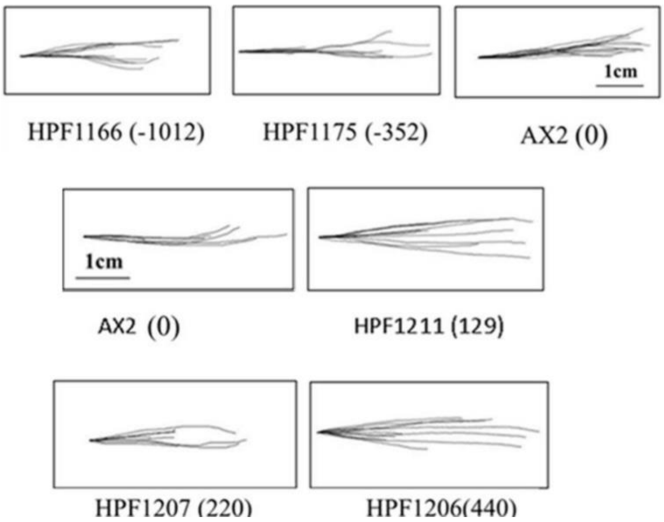

Supplementary Figure S3. Phototaxis by slugs of *D. discoideum* DJ-1 transformants with altered DJ-1 expression. The slug trails of AX2 and DJ-1 transformants are shown migrating to the light source which is to the right of the figures. HPF1175 and HPF1166 are antisense-inhibited transformants and the negative numbers in the brackets are the copy numbers of the antisense construct pPROF688, the negative numbers being used by convention to indicate the resulting reduction in expression. HPF1211, HPF1207 and HPF1206 are DJ-1 overexpression transformants and the numbers in the brackets are the copy numbers of the overexpression construct pPROF690. From Figure 4 of Chen *et al*. [2].

Supplementary Figure S4.

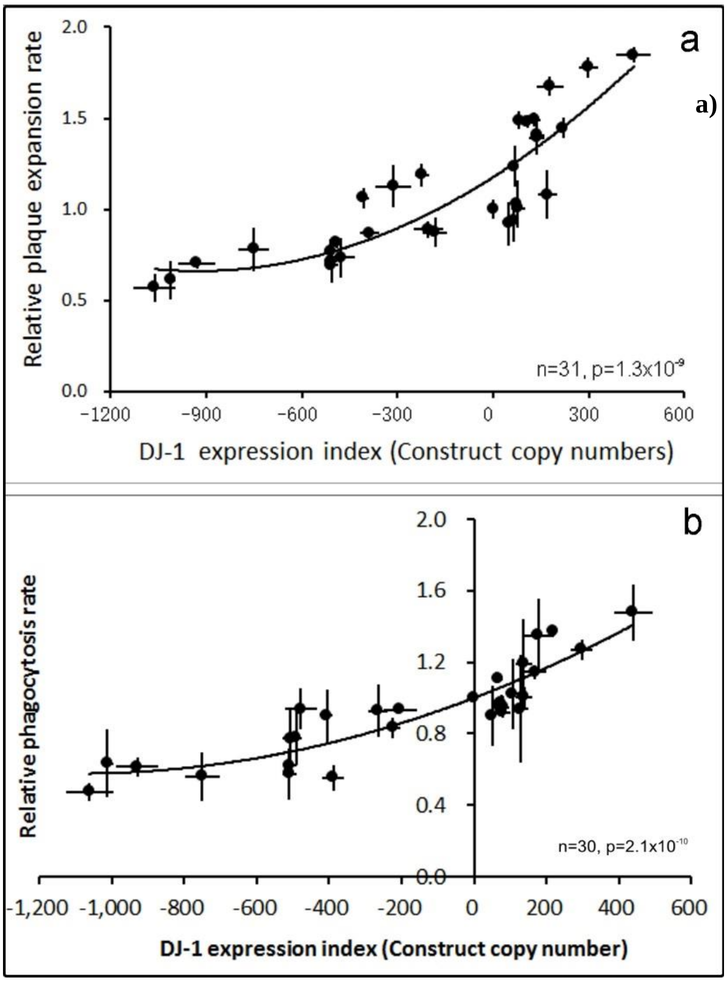

Supplemental Figure S4. DJ-1 upregulates growth on bacterial lawns and phagocytosis.

a) The plaque expansion rates on *E. coli* B2 lawns for the transformants were measured and normalized against the wild type parent, AX2, then plotted against the DJ-1 expression index. Each point (± standard error) represents the mean from 4 independent experiments, each of which involved duplicate assays. The regression was fitted by least squares to a quadratic polynomial and was highly significant (F test).

The phagocytosis rates of the wild type AX2 and the transformants with altered DJ-1 expression were measured and normalized against AX2. Each point represents the mean from 3 independent experiments, each of which included duplicate measurements. The regression was highly significant (F test). Error bars are standard errors of the mean.

From Figure 5 of Chen *et al*. [2].

Supplementary Figure S5.

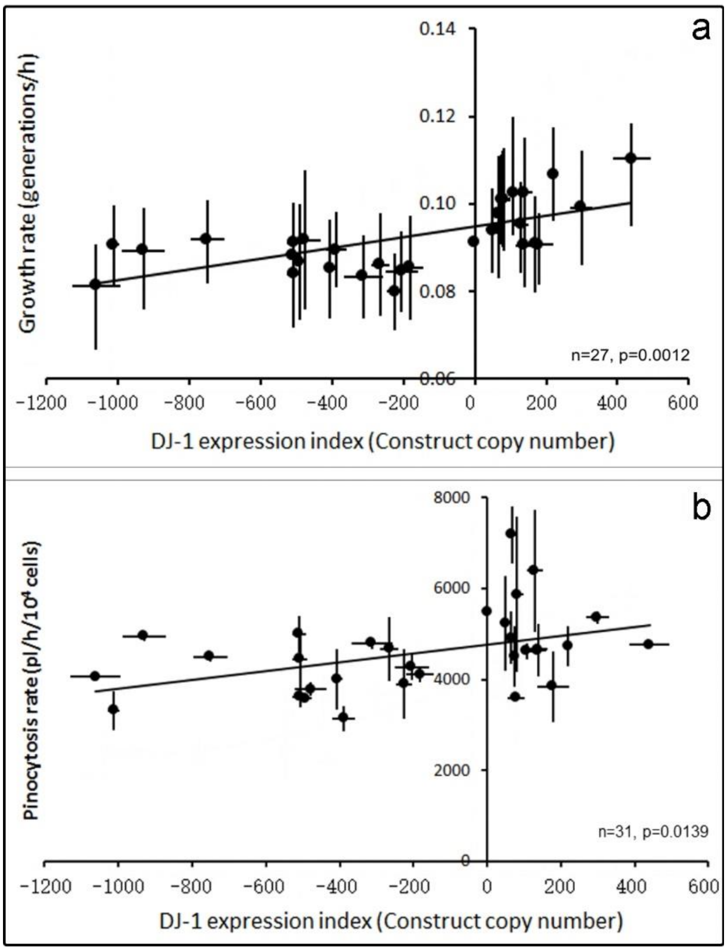

Supplementary Figure S5. DJ-1 modestly upregulates growth in liquid medium and pinocytosis.

a) The growth rate (inverse of the doubling time) during exponential growth of DJ-1 transformants was measured and compared with the parent strain, AX2. The linear regression was highly significant (F test). Error bars are standard errors of the mean from 3 independent experiments.

AX2 and transformants were grown in low fluorescence HL-5 medium containing FITC-dextran. The pinocytosis of the transformants increased as the DJ-1 expression levels were increased. The linear regression was statistically significant (F test). Error bars are standard errors of the mean from 3 independent experiments.

From Figure 6 of Chen *et al*. [2].

Supplementary Figure S6

Supplementary Figure S6. DJ-1 knockdown causes aberrant *Dictyostelium* morphogenesis.

a: The fruiting bodies of DJ-1 antisense-inhibited transformants. Strain names are indicated by HPF numbers and the negative numbers in brackets represent the copy number of pPROF688 (antisense-inhibition construct). The spore droplets (sori) are enlarged, while the stalks are thicker and shorter in the DJ-1 antisense-inhibited strains. The severity of the defect correlates with the reduction in DJ-1 expression.

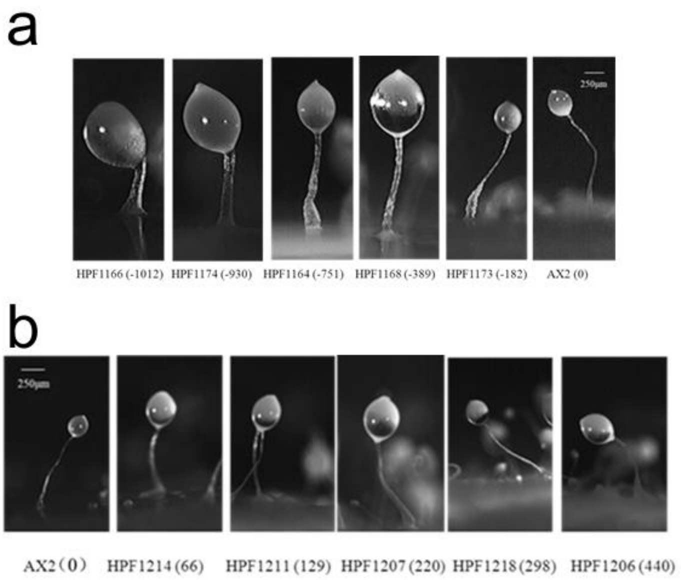

b: The fruiting bodies of DJ-1 overexpression transformants. Strain names are indicated by HPF numbers and the numbers in brackets represent the copy number of pPROF690 (overexpression construct). Wild type AX2 fruiting bodies contain long slender stalks as do the transformants overexpressing DJ-1.

From Figure 7 of Chen *et al*. (2017) [2].

Supplementary Table S1.

Phagocytosis Multiple Regression

X-variables:
x_1=DA copies
x_2=DA copies^2
x_3=DA/H_2O_2 Intercept (Dummy 1)
x_4=DA/H_2O_2 Slope (Dummy 2)
x_5=DAA/H_2O_2 Intercept (Dummy3)
x_6=DAA H_2O_2 slope (Dummy 4)
x_7=AA copies

Y-Variable: Relative phagocytosis rate

Method: Backward

Steps	P	R-Square	Corrected
x_6 (DAA H_2O_2 slope) (Dummy 4) (-)	0.85814	0.711994	0.657993
x_7 (AA copies) (-)	0.559487	0.708863	0.664752
x_4 (DA/H_2O_2 Slope) (Dummy 2) (-)	0.373351	0.701678	0.666581
x_5 (DAA/H_2O_2 Intercept) (Dummy3) (-)	0.402254	0.695365	0.669254
x_2 (DA copies^2) (-)	0.17552	0.678725	0.660877

Summary

	N	R	R-Square	Std.Error
**normal**	39	0.823848	0.678725	0.103116
**corrected**		0.812943	0.660877	

Equation

	Coefficient	95% Conf.	Std.Error	T	P
Constant	0.932557	0.060662	0.02991	31.17853	1.2E-27
x_1 (DA copies)	-0.00039	0.000113	5.56E-05	-6.96495	3.65E-08
x_3 (DA/H_2O_2 Intercept) (Dummy 1)	-0.13387	0.072513	0.035754	-3.74434	0.000631

Analysis of variance

	Sum of	Degrees of	Mean	F	P
**Regression**	0.808676	2	0.404338	38.02681	1.33E-09
**Residue**	0.382787	36	0.010633		
**Total**	1.191464	38	0.031354		

Supplementary Table S1. Multiple regression analysis of effects on phagocytosis rates of DJ-1 knock down, DJ-1/AMPK double knock down and oxidative stress.
A multiple regression analysis [3] was conducted of the data presented in Figure 5 using WinStat (a Microsoft Excel plugin). The initial model included as x variables the number of copies of the DJ-1 antisense construct (DA copies) and the number of copies of the AMPK antisense construct (DAA copies), the square of the number of copies of the DJ-1 antisense construct (DA copies^2), and dummy variables allowing the intercepts (Dummy1 and Dummy3) and slopes (Dummy2 and Dummy4) in the presence of H_2O_2 to differ amongst the strains. The Y variable was the normalized rate of phagocytosis.

Initial model:

$Y = b_o + b_1x_1 + b_2x_2 + b_3x_3 + b_4x_4 + b_5x_5 + b_6x_6 + b_7x_7$

Where

x_1 = Number of copies of the DJ-1 antisense construct

$x_2 = x_1^2$ provides a quadratic rather than linear fit (if b_2 significantly different from 0)

x_3 = 1 for DJ-1 antisense (DA) strains with H_2O_2, otherwise 0. Allows the intercept to be different for DA strains with H_2O_2 (if b_3 significantly different from 0).

$x_4 = x_1$ for DJ-1 antisense (DA) strains with H_2O_2, otherwise 0. Allows the slope to be different for DA strains

with H_2O_2 (if b_4 significantly different from 0).
$x_5 = 1$ for cotransformants (DAA) strains with H_2O_2, otherwise 0. Allows the intercept to be different for DAA strains with H_2O_2 (if b_5 significantly different from 0).
$x_6 = x_1$ for cotransformants (DAA) strains with H_2O_2, otherwise 0. Allows the slope to be different for DAA strains with H_2O_2 (if b_6 significantly different from 0).
x_7 = Number of copies of AMPK antisense in cotransformants (AA copies), otherwise 0. Detects if there is a significant correlation (if b_7 significantly different from 0) with the copy number of the AMPK antisense construct (not plotted).
The analysis included all x variables initially and then progressively removed nonsignificant variables (see listed Steps - null hypothesis $b_i = 0$ accepted, $p > 0.05$) until only those x variables remained whose coefficients (b_i) were significantly different from zero. The final model retained only the DJ-1 antisense copy number (x_1) and the presence of H_2O_2 in the case of the DJ-1 antisense strains (x_3) as being significant variables for the phagocytosis rates. As can be seen in the corresponding plot (Fig. 5), the presence of H_2O_2 reduced phagocytosis rates significantly (b_3 significantly different from 0) and by the same amount (b_4 not significantly different from 0) in all DJ-1 antisense strains. This effect was reversed by the presence of the AMPK antisense construct, regardless of DJ-1 copy number (b_5 and b_6 not significantly different from 0).

Supplementary Table S2.

Generation Time Multiple Regression

X-variables: x_1=DA copies
x_2=H_2O_2 Intercept (Dummy 1)
x_3=H_20_2 Slope (Dummy 2)
x_4=DAA intercept (Dummy 3)
x_5=DAA slope (Dummy 4)
x_6=AA copies

Y-Variable: Generation time (h)

Method: Backward

**Steps**	**P**	**R-Square**	**Corrected**
x_6 (AA copies) (-)	0.851669	0.73618	0.700529
x_1 (DA copies) (-)	0.667399	0.734842	0.706931
x_4 (DAA intercept) (Dummy 3) (-)	0.376609	0.729257	0.708431

Summary

	**N**	**R**	**R-Square**	**Std.Error**
**normal**	43	0.853965	0.729257	1.946384
**corrected**		0.841683	0.708431	

Equation

	**Coefficient**	**±95% Conf.**	**Std.Error**	**T**	**P**
Constant	11.53286	1.052197	0.520193	22.17034	1.03E-23
x_2 (H_2O_2 Intercept) (Dummy 1)	1.68457	1.598734	0.790394	2.131304	0.039422
x_3 (H_20_2 Slope) (Dummy 2)	-0.01306	0.00363	0.001795	-7.27531	9E-09
x_5 (DAA slope) (Dummy 4)	0.008602	0.004649	0.002299	3.742145	0.000587

Analysis of variance

	**Sum of**	**Degrees of**	**Mean**	**F**	**P**
**Regression**	397.9653	3	132.6551	35.01602	3.77E-11
**Residue**	147.7481	39	3.788412		
**Total**	545.7134	42	12.99318		

Supplementary Table S2. Multiple regression analysis of generation time for DJ-1 antisense and DJ-1/AMPK double antisense transformants in the presence of H_2O_2.
Multiple regression analysis [3] was conducted on the generation time data presented in Figure 6a using WinStat (a Microsoft Excel plugin). To facilitate comparisons across a similar range of DJ-1 copy numbers, the analysis used data for strains with up to about 900 copies of the DJ-1 antisense construct. The initial model included as x variables the number of copies of the DJ-1 antisense construct (DA copies) and the AMPK antisense construct (AA copies), and dummy variables allowing the intercepts and slopes amongst the strains to differ in the

presence of H_2O_2 with or without the AMPK antisense construct. The y variable was the generation time (h).
Initial model:
$Y = b_o + b_1x_1 + b_2x_2 + b_3x_3 + b_4x_4 + b_5x_5 + b_6x_6 + b_7x_7$
Where
x_1 = Number of copies of the DJ-1 antisense construct
x_2 = 1 for DJ-1 antisense (DA) strains with H_2O_2, otherwise 0 (allows the intercept to differ in the presence of H_2O_2)
$x_3 = x_1$ for DJ-1 antisense (DA) strains with H_2O_2, otherwise 0 (allows the slope to differ in the presence of H_2O_2)
x_4 = 1 for cotransformants (DAA) strains with H_2O_2, otherwise 0 (allows the intercept to differ in the presence of H_2O_2 and the AMPK antisense construct)
$x_5 = x_1$ for cotransformants (DAA) strains with H_2O_2, otherwise 0 (allows the slope to differ in the presence of H_2O_2 and the AMPK antisense construct)
x_6 = Number of copies of AMPK antisense in cotransformants, otherwise 0 (allows the slope to differ in proportion to the number of copies of the AMPK antisense construct)
The analysis included all x variables initially and then progressively removed nonsignificant (null hypothesis $b_i = 0$ accepted at $p > 0.05$) variables until only those x variables remained whose coefficients (b_i) were significantly different from zero. The final model retained the intercept for the DJ-1 antisense strains (Constant = b_0), the difference between this and the intercept in the presence of H_2O_2 (b_2), the slope in the presence of H_2O_2 (b_3) and the change in the slope in cotransformants (b_5) as being significant coefficients for the regression. As can be seen in the corresponding plot (Figure 6a), the presence of H_2O_2 prolonged the generation times significantly and this effect was dramatically enhanced in a copy number-dependent manner by the presence of the DJ-1 antisense construct in a copy number-dependent manner. The presence of the AMPK antisense construct in the same cells reduced the impact of the DJ-1 knock down (but this did not show a significant dependence on the number of copies of the AMPK antisense construct). The presence of the AMPK antisense construct did not exert a significant effect on the effect of H_2O_2 on generation time when DJ-1 was expressed at wild type levels (intercept - construct copy number 0).

Supplementary Table S3.

Lag Time Multiple Regression

X-variables: x_1=DA copies
x_2=DA
x_3=DAA intercept
x_4=DAA slope (Dummy 2)
x_5=AA copies
x_6=AA
x_7=DAA

Y-Variable: lag phase

Method: Backward

Steps	P	R-Square	Corrected
x_5 (AA copies) (-)	0.816874542	0.85037998	0.80957452
x_6 (AA copies^2) (-)	0.510766727	0.84734068	0.81415387
x_3 (DAA intercept) (Dummy1) (-)	0.278914315	0.83917829	0.81237467

Summary

	N	R	R-Square	Std.Erro
**normal**	29	0.91606675	0.83917829	5.711255
**corrected**		0.90131830	0.81237467	

Equation

	Coefficient	±95% Conf.	Std.Error	T	P
Constant	24.62161883	4.85568583	2.35267061	10.46539	2.00702E-
x_1 (DA copies)	-0.095167476	0.03499557	0.01695600	-5.61261	8.89658E-
x_2 (DA copies^2)	-7.42556E-05	5.4908E-05	2.6604E-05	-2.791151	0.01013525
x_4 (DAA slope) (Dummy 2)	0.089982789	0.04451401	0.02156787	4.172076	0.00034081
x_7 (DAA slope^2)	0.000103117	8.00723E-05	3.87965E-	2.657887	0.01377061

Analysis of variance

	Sum of	Degrees of	Mean	F	P
**Regression**	4084.924026	4	1021.23100	31.3084	3.31352E-
**Residue**	782.8425053	24	32.6184377		
**Total**	4867.766531	28	173.848804		

Supplementary Table S3. Multiple regression analysis of lag time for DJ-1 antisense and DJ-1/AMPK double antisense transformants in the presence of H_2O_2.
Multiple regression analysis [3] of the lag phase data presented in Figure 6b was conducted using WinStat (a

Microsoft Excel plugin). To facilitate comparisons across a similar range of DJ-1 copy numbers, the analysis used data for strains with up to about 900 copies of the DJ-1 antisense construct. Because the experiment begins with cells already growing exponentially in HL-5 medium, the lag time was zero in all cases in the absence of H_2O_2 (and so is not included). As x variables, the initial regression model included the number of copies of the DJ-1 antisense construct (DA copies) and the AMPK antisense construct (AA copies), the squares of these copy numbers (to allow fitting quadratic polynomials) and dummy variables allowing the intercepts and slopes amongst the strains to differ with (DAA, cotransformants) or without (DA, DJ-1 antisense transformants) the AMPK antisense construct. The y variable was the length of the lag phase (h).

Initial model:

$Y = b_o + b_1x_1 + b_2x_2 + b_3x_3 + b_4x_4 + b_5x_5 + b_6x_6 + b_7x_7$

Where

x_1 = Number of copies of the DJ-1 antisense construct

$x_2 = x_1^2$ (to fit a quadratic polynomial)

x_3 = 1 for cotransformants (DAA), otherwise 0 (allows the intercept to differ in the cotransformants)

$x_4 = x_1$ for cotransformants (DAA), otherwise 0 (allows the slope to differ in the cotransformants)

x_5 = Number of copies of the AMPK antisense construct in cotransformants, otherwise 0 (allows the lag time to vary in proportion to the number of copies of the AMPK antisense construct)

$x_6 = x_5^2$ (allows the lag time to vary in proportion to the square of the number of copies of the AMPK antisense construct ie as a quadratic polynomial)

$x_7 = x_2$ for cotransformants (DAA), otherwise 0 (allows the coefficient for the x^2 component of a fitted quadratic polynomial to differ in the cotransformants)

The analysis included all x variables initially and then progressively removed nonsignificant (null hypothesis $b_i = 0$ accepted at $p > 0.05$) variables until only those x variables remained whose coefficients (b_i) were significantly different from zero. The final model retained as significant ($p < 0.05$) the intercept and both slope coefficients for the DJ-1 construct antisense copy number (b_o, b_1, b_2) showing that DJ-1 knock down significantly lengthens the lag phase and the relationship between the lag time and the DJ-1 construct copy number was significantly better represented by a quadratic equation (shown as the blue line in Figure 10d) than by a straight line. Also retained as significant at $p < 0.05$ were the differences in the slope coefficients (b_4 and b_7) between cotransformants (DAA) and the DJ-1 transformants (DA). The resulting regression line for the cotransformants did not differ significantly from a straight line (95% confidence interval for the slope, b_2+b_7, overlaps 0). As can be seen in the corresponding plot (Figure 6b), the presence of H_2O_2 increased lag times significantly (from 0) and this effect was dramatically enhanced by the presence of the DJ-1 antisense construct in a copy number-dependent manner. The presence of the AMPK antisense construct in the same cells reduced the impact of the DJ-1 knock down (but the magnitude of this effect was not significantly dependent on the copy number of the AMPK antisense construct, variables x_5 and x_6). The presence of the AMPK antisense construct did not exert a significant effect on the effect of H_2O_2 on the lag time when DJ-1 was expressed at wild type levels (intercept - construct copy number 0).

Supplementary References

[1] Lay, S.T.; Sanislav, O.; Annesley, S.J.; Fisher, P.R. Mitochondrial stress tests using Seahorse respirometry on intact *Dictyostelium discoideum* cells. *Methods Mol Biol* 2016, *1407*, 41-62;

[2] Chen, S.; Annesley, S.J.; Jasim, R.A.F.; Musco, V.J.; Sanislav, O; Fisher, P.R. The Parkinson’s disease-associated protein DJ-1 plays a positive nonmitochondrial role in endocytosis in *Dictyostelium* cells. *Disease Models and Mechanisms* 2017, *10*, 1261-1271.

[3] Neter, J.; Wasserman, W. *Applied linear statistical models.* 1974. Richard D. Irwin Inc., Homewood, Illinois, USA. ISBN 0-256-01498-1.
